# The Interplay between GSK3β and Tau Ser262 Phosphorylation during the Progression of Tau Pathology

**DOI:** 10.3390/ijms231911610

**Published:** 2022-10-01

**Authors:** Liqing Song, Daniel E. Oseid, Evan A. Wells, Anne Skaja Robinson

**Affiliations:** 1 Department of Chemical Engineering, Carnegie Mellon University, Pittsburgh, PA 15213, USA; 2 Tulane Brain Institute, Tulane University, New Orleans, LA 70118, USA

**Keywords:** tauopathies, Alzheimer’s disease, neurodegeneration, hyperphosphorylation, accumulation, Ser262, GSK3β, exosomes, oligomerization, secretion, seeding activity, transcellular propagation, β-amyloid

## Abstract

Tau hyperphosphorylation has been linked directly to the formation of toxic neurofibrillary tangles (NFTs) in tauopathies, however, prior to NFT formation, the sequence of pathological events involving tau phosphorylation remains unclear. Here, the effect of glycogen synthase kinase 3β (GSK3β) on tau pathology was examined independently for each step of transcellular propagation; namely, tau intracellular aggregation, release, cellular uptake and seeding activity. We find that overexpression of GSK3β-induced phosphorylated 0N4R tau led to a higher level of tau oligomerization in SH-SY5Y neuroblastoma cells than wild type 0N4R, as determined by several orthogonal assays. Interestingly, the presence of GSK3β also enhanced tau release. Further, we demonstrated that cells endocytosed more monomeric tau protein when pre-phosphorylated by GSK3β. Using an extracellular vesicle (EVs)-assisted tau neuronal delivery system, we show that exosomal GSK3β-phosphorylated tau, when added to differentiated SH-SY5Y cells, induced more efficient tau transfer, showing much higher total tau levels and increased tau aggregate formation as compared to wild type exosomal tau. The role of a primary tau phosphorylation site targeted by microtubule-affinity regulating kinases (MARKs), Ser262, was tested by pseudo-phosphorylation using site-directed mutagenesis to aspartate (S262D). S262D tau overexpression significantly enhanced tau release and intracellular tau accumulation, which were concurrent with the increase of pathological states of tau, as determined by immunodetection. Importantly, phosphorylation-induced tau accumulation was augmented by co-transfecting S262D tau with GSK3β, suggesting a possible interplay between Ser262 phosphorylation and GSK3β activity in tau pathology. Lastly, we found that pre-treatment of cells with amyloid-β (Aβ) further tau phosphorylation and accumulation when Ser262 pre-phosphorylation was present, suggesting that S262 may be a primary mediator of Aβ-induced tau toxicity. These findings provide a potential therapeutic target for treating tau-related disorders by targeting specific phospho-tau isoforms and further elucidate the GSK3β-mediated pathological seeding mechanisms.

## 1. Introduction

Tauopathies, including Alzheimer’s Disease (AD), are characterized by the formation of toxic neurofibrillary tangles (NFTs), composed of insoluble tau aggregates, that drive the progression of neurodegeneration [1]. More than 45 phosphorylation sites have been detected on insoluble aggregated tau or NFTs [1], indicating a pathological role of hyper-phosphorylated tau in the formation of NFTs. Abnormal hyperphosphorylation of tau appears to result from the activity of multiple tau kinases such as glycogen synthase kinase 3β (GSK-3β), cyclin-dependent kinase 5 (CDK5), and cAMP-dependent protein kinase (PKA) [2,3]. A higher amount of non-microtubule (MT) associated (free) tau likely provides the essential component for forming toxic tau seeds that propagate tau pathology during the progression of neurodegeneration. Transcellular tau propagation has been implicated in tauopathies following a ‘prion-like’ transmission pattern [4], involving the release of intracellular pathological tau into the extracellular space, uptake of tau seeds by the recipient cells, and the formation of new intracellular aggregates in the recipient cells [5]. These tau seeds, such as soluble tau aggregates or oligomeric tau, may be the most toxic and pathologically significant tau species [6,7,8,9] that are responsible for initiating tau cellular propagation. Despite efforts to identify the role of phosphorylation in forming NFTs or insoluble tau aggregates, the steps in the tau phosphorylation cascade that affect the formation of tau oligomers and, therefore, pathological tau propagation have remained less clear.

GSK3β is proline-directed serine/threonine kinase (i.e., SP/TP sites) that phosphorylates tau at more than 42 sites associated with AD [10], such as Tyr18, Thr181, Ser199, Ser202, Thr205, Thr217, Thr231, Ser396 and Ser422 [2]. GSK3β activity is upregulated in AD brain [11], and this activity changes dynamically as the disease progresses. Of note, a previous study characterized the temporal phosphorylation profiles of several sites phosphorylated by GSK3β, showing increased phosphorylation at several residues including Tyr18, Thr231 Ser199, Ser202/Thr205, and Ser422 at increasing Braak stages throughout the entire isocortex and transentorhinal cortex [12]. Those findings implied a causative relationship between the degree of tau phosphorylation and disease progression. However, less is known about how GSK3β-induced tau phosphorylation enables tau propagation due to limited investigation to date [13,14]. In situ tau phosphorylation by GSK3β or pseudo-phosphorylation at GSK3β-phosphorylating sites increased the levels of tau in the culture medium as compared to wild type tau in Hela cells [13], HEK293 cells, and SH-SY5Y neuroblastoma cells [14], suggesting phosphorylation may be a major driving force for tau secretion to the extracellular matrix and subsequent spread of tau pathology [14]. The relevance of tau phosphorylation for cellular uptake was also evaluated using brain extracts from tau-transgenic mice models. rTg4510 mice, overexpressing human P301L tau, showed more human tau immunoreactivity than that of rTg21221 mice, which overexpress wild type human tau. Biochemical characterization of brain extracts from rTg4510 mice showed higher levels of phosphorylation as compared to those of rTg21221 [15], implicating phosphorylation in pathology and transmission. Elevated tau oligomer levels correlated with a higher level of tau phosphorylation in the AD brain, revealing a possible relationship between tau phosphorylation and oligomerization [16].

Recently, abnormal tau phosphorylation by microtubule-affinity regulating kinases (MARKs) has gained growing interest [17] due to their crucial effects on inducing early tau phosphorylation during AD neurodegeneration. MARKs are non-SP/TP kinases, phosphorylating KXGS motifs in the repeat domain of tau located in the microtubule-binding region such as Ser262 and Ser356 [18]. In AD brain, protein expression of MARK isoforms, MARK3 and MARK4, were found to be elevated and this expression was highly correlated with Ser262 phosphorylation in granulovacuolar degeneration bodies (GVDs) of AD in a Braak-stage dependent manner [19]. Phosphorylation of Ser262 triggers microtubule disruption, resulting in morphological changes in cells and, eventually, cell death [20]. A recent study reported a possible correlation between Ser262 phosphorylation and the aggressiveness of clinical disease state [16]. Although Ser262 phosphorylation has shown relevance to tau toxicity, the molecular mechanisms need further investigation.

Based on previous studies, we hypothesized that tau phosphorylation induced by GSK3β is actively involved in every step of the tau transcellular propagation process, leading to a higher level of toxic tau oligomers. Moreover, Ser262 phosphorylation is one of the earliest occupied phosphorylation sites found in AD, which is much earlier than the phosphorylation sites on tau formed by GSK3β [21]. Given the sequential ordering between Ser262 phosphorylation by MARK and GSK3β action on tau, we also sought to determine how the presence of phosphorylated Ser262 affects the activity of GSK3β, thus leading to the subsequent pathological events. To test this, we first broke down transcellular tau propagation into three steps involving tau release, uptake, and subsequent intracellular aggregation, and examined the effects of tau phosphorylation on each step independently. Next, the effect of phosphorylation on the spread of tau pathology was validated using an established tau transcellular transmission model. Furthermore, the pathological consequences of tau phosphorylation by Ser262 phosphorylation were investigated. Lastly, the dependency of GSK3β activity on Ser262 phosphorylation was evaluated. Findings from our study provide a mechanistic basis for Ser262 phosphorylation-induced toxicity, which presents a potential target for treating tau-related neurodegeneration.

## 2. Results

### 2.1. Phosphorylation Characteristics of PS19 Transgenic Mice

**Figure 1:**
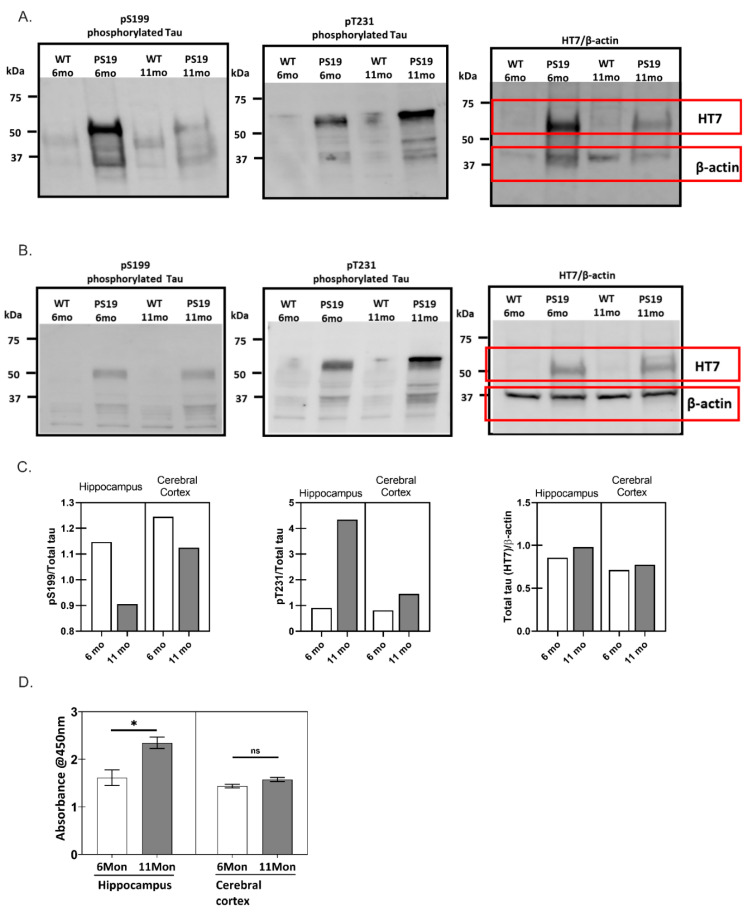
The phosphorylation characteristics of PS19 transgenic mice brain extracts. Immunoblot of phosphorylated tau at Ser199 (pS199), Thr231 (pT231), and total tau (HT7) detected in the crude cell lysate of 6- and 11-month-old wild type (WT) and P301S (PS19) mice hippocampus (**A**) and cerebral cortex (**B**). (**C**) Quantification of the relative ratio of tau markers, pS199 and pT231, to total tau for PS19 mice hippocampus and cerebral cortex at 6- and 11-month-old (normalized to the average level in 6-month-old PS19 mice counterpart, which was set to 1. Total tau level was normalized to an endogenous control, β-actin. (**D**) The oligomeric tau content in crude cell lysates from 6- and 11-month-old PS19 mice hippocampus and cerebral cortex was measured and quantified by ELISA assay (* represents *p* < 0.05 between average of different groups, *n* = 3; ns = not significant).

The spread of tau pathology in the brain starts in the entorhinal cortex and spreads anatomically in a defined pattern to the hippocampus, dentate gyrus, and finally to the neocortex [22]. The phosphorylation profile of tau was characterized from hippocampal and cerebral cortical brain samples of PS19 mice, a P301S tauopathy mouse model [23] (Figure 1A,B). This model displays human tau pathology and has been validated to recapitulate the development of neurodegeneration and diverse pathological phenotypes, including gliosis, synaptic loss, tangles, and neuronal loss [22]. Western analysis was performed to compare the levels of phosphorylated tau at Ser199 and Thr231 and total tau, using immunoblotting against the total tau antibody HT7 across different brain samples from PS19 mice. Hippocampal (Figure 1A) and cerebral cortical brain samples (Figure 1B) showed similar phosphorylation patterns, indicating that tau pathology may have progressed to the cerebral cortex of the PS19 brain as early as six months. The level of tau phosphorylation at both sites, Ser199 and Thr231, was higher for both six- and 11-month samples of PS19 brain as compared to their healthy (WT) counterparts, consistent with the involvement of phosphorylated tau in the disease progression. Interestingly, phosphorylation at Ser199 in hippocampal brain samples of PS19 mice decreased in mice aged six months to 11 months (Figure 1C). As an early tau phosphorylation site, Ser199 phosphorylation precedes the formation of insoluble NFTs [24], while a reduction in its phosphorylation in the detergent-soluble fraction suggested an increase in the formation of insoluble tau fibrils in aging PS19 mice. Additionally, phosphorylation at site Thr231 increased from six months to 11 months as the content of oligomeric tau increased, as quantified by ELISA (Figure 1D). These results are consistent with a previous study [12] that demonstrated that the phosphorylation at Thr231 increased at increasing Braak stages throughout the entire isocortex and transentorhinal cortex. A sequential phosphorylation process—phosphorylation at Thr231 preceding the formation of tau oligomers, while tau oligomerization occurred before phosphorylation at Ser202/Thr205—was previously reported in SH-SY5Y human neuroblastoma cells [8]. Further, phosphorylation at PHF-1-targeted epitopes, Ser396/Ser404, facilitates the formation of insoluble tau aggregates as demonstrated in a split GFP complementation assay [25]. Taken together, the possible correlation between phosphorylation at Thr231 and tau oligomer formation during the disease progression observed in our study suggests a possible role of Thr231 phosphorylation in initiating and promoting tau oligomerization and led to our further investigation of tau phosphorylation.

### 2.2. The Role of GSK3β-Induced Phosphorylation in Tau Oligomerization and Secretion

**Figure 2:**
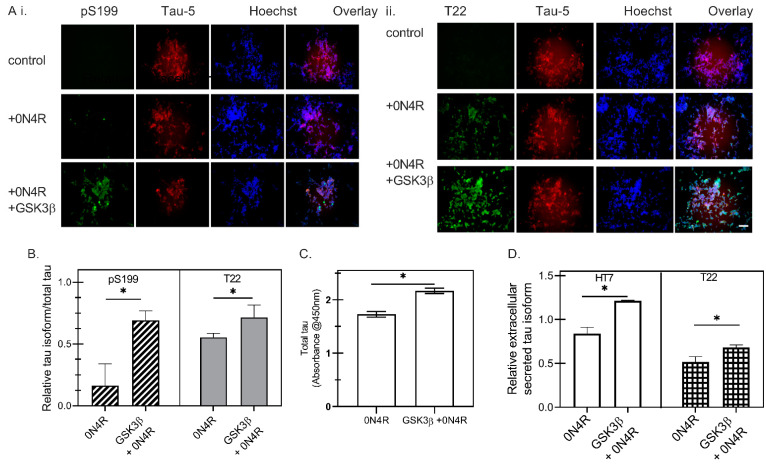
The phosphorylation characteristics of PS19 transgenic mice brain extracts. Characterization of SH-SY5Y cells transfected with wild type 0N4R and GSK3β constructs. Representative fluorescent images of tau markers, including pS199 (Green)/Tau-5 (Red)/Hoechst (Blue) (**A**(**i**)) and T22(Green)/Tau-5 (Red)/Hoechst (Blue) (**A**(**ii**)) for SH-SY5Y cells three days post-transfection. Scale bar = 100 µm. (**B**) Quantitative analysis of fluorescence intensity of pS199 (dark gray hatched bars) and T22 (light gray solid bars), normalized to Tau-5+ intensity as a measure of the total tau expression, for SH-SY5Y cells expressing 0N4R tau with GSK3β co-transfection (GSK3β + 0N4R) or without GSK3β (0N4R), where * represents *p* < 0.05 for *n* = 3. (**C**) The oligomeric and total tau were measured by sandwich ELISA of SH-SY5Y cell lysates using T22 and human tau antibody, respectively. * *p* < 0.05 for 0N4R compared with GSK3β + 0N4R (*n* = 3). (**D**) Total tau (open bars) or oligomeric tau (boxes-filled bars) of the conditioned medium were measured by indirect ELISA using HT7 and T22, respectively, to investigate the effects of phosphorylation on tau secretion. (* represents *p* < 0.05 for 0N4R compared with GSK3β + 0N4R (*n* = 3)).

To introduce phosphorylation on tau, wild type 0N4R was phosphorylated by transiently overexpressing glycogen synthase kinase 3β (GSK3β) in different cellular models, SH-SY5Y cells (Figure 2) and HEK293 cells (Supplementary Figure S1). We observed that co-transfection of 0N4R with GSK3β led to an increase in the level of phosphorylation as determined by immunostaining cells with a phospho-tau antibody (pS199, a tau phosphorylation site reported in both healthy and AD brains [26]) (Figure 2A(i),B). To evaluate the oligomeric content of different cell samples, we carried out immunostaining (Figure 2A(ii)) against tau oligomeric tau antibody T22 and total tau antibody, Tau5, showing an increase in the level of oligomers formed in the presence of co-expressed GSK3β. The increase of oligomeric tau was also confirmed by sandwich ELISA, in which oligomeric and total tau in cell lysates were measured using T22 and human tau antibody, respectively (Figure 2C), and a modest increase was observed in GSK3β co-expressing cells. 

A previous study reported that co-transfection of GSK3β led to an increased level of secreted tau, suggesting that phosphorylation may be a major driving factor for tau secretion [14]. In our study, we evaluated oligomeric tau levels as well as total tau present in the culture supernatant, via indirect ELISA with T22 and HT7 antibodies to further investigate the effects of phosphorylation on tau secretion (Figure 2D). We find here that co-transfection of GSK3β increased the total tau secretion (Figure 2D), consistent with the earlier study. We also determined that oligomeric tau secretion (Figure 2D) into the cell culture medium increased to a modest but statistically significant extent with GSK3β co-transfection, which suggests that phosphorylation contributes to the increased secretion of tau oligomers as well as total extracellular tau.

### 2.3. The Effect of Phosphorylation on Tau Seeding Activity

#### 2.3.1. Phosphorylation Characterization of *E. coli*-Expressed Tau with GSK3β Co-Expression

**Figure 3:**
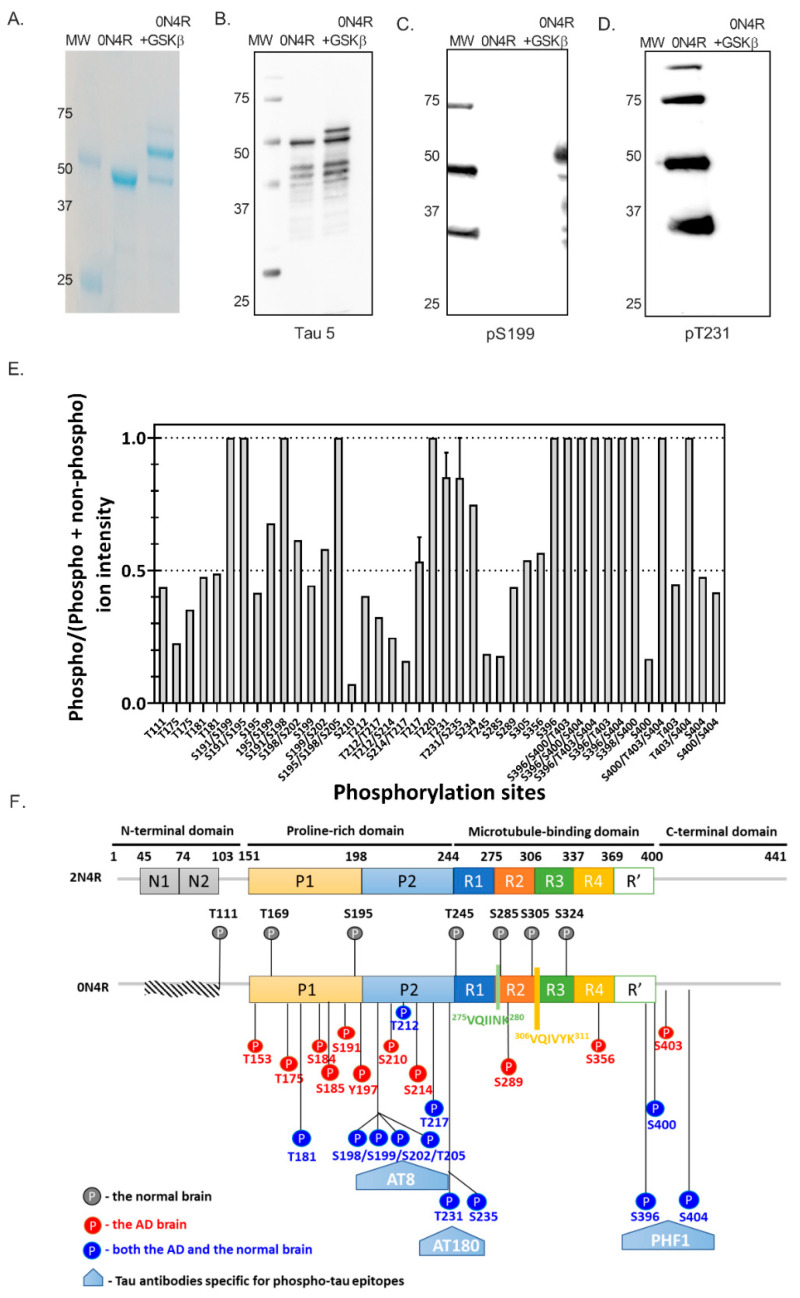
Co-expression of tau with GSK3β phosphorylates 0N4R tau in *E. coli*. (**A**) Coomassie-stained SDS-PAGE of purified non-phosphorylated and phosphorylated tau (2 μg each). Western blot analysis using Tau-5 (**B**), anti-phosphoTau-S199 (**C**), and anti-phosphoTau-T231 (**D**) antibodies. Protein Western C ladder (Bio-Rad) standards are indicated (kDa). (**E**) Quantitative analysis of phosphorylation at 29 phosphorylation sites, as determined by LC-MS/MS analysis of purified phosphorylated tau (*n* = 2). (**F**) Schematic illustration of 29 phosphorylation sites on tau protein and epitopes specific for major tau antibodies. Red color denotes amino acids phosphorylated in the AD brain, blue in both AD and normal brain, and grey in the normal brain. Tau antibodies AT8, AT180, and PHF-1 specific for phospho-tau epitopes are shown in white letters, in a shape colored with light blue: AT8 (pSer202 and pThr205), AT180 (pT231), and PHF-1 (pSer396 and pSer404). The residues are numbered based on the amino acid designations for the full-length tau isoform, 2N4R.

Intracellular tau oligomerization formed following the uptake of recombinant wild type 0N4R tau or phosphorylated tau was evaluated to determine whether tau phosphorylation contributed to cellular tau aggregation. For expressing recombinant tau proteins, 0N4R tau DNA was cloned into a pETM-13 expression vector and transformed by heat shock into chemically competent Rosetta™ (DE3) cells using standard techniques. To obtain phosphorylated tau monomer, GSK3β was cloned into a pBAD expression vector for co-expression. Although several kinases have been implicated in tau phosphorylation, we chose GSK3β as an initial modification agent, as previous reports suggested a hierarchical process that starts with GSK3β [10]. Non-phosphorylated and phosphorylated 0N4R tau were isolated as described in Methods, yielding >95% purified protein, as determined by Coomassie staining (Figure 3A). The apparent molecular weight of non-phosphorylated tau was approximately 48 kDa (Figure 3A), consistent with earlier reports [27]. The phosphorylated tau showed two predominant bands at 48 kDa and 52 kDa (Figure 3A), which indicates that samples of purified phosphorylated tau contain small amounts of partially phosphorylated tau protein. Here, Western analysis confirmed the identity of tau (Figure 3B), and phosphorylation of both bands in the phospho-tau species was confirmed with anti-phospho tau antibodies at Ser199 and Thr231 (Figure 3C,D).

Liquid chromatography combined with tandem mass spectrometry (LC-MS/MS) was used to identify phospho-peptides following tryptic digestion and their sites of phosphorylation (performed by Creative Proteomics). Tau protein was efficiently hyper-phosphorylated by co-expression with GSK3β, with 29 phosphorylation sites identified (Figure 3E). Among all the detected sites, only 7 out of 29 phosphorylated amino acids are present in the normal human brain samples [26]; the other detected phosphorylation sites on tau protein have been associated with AD brain (Figure 3E,F). A significant number of phosphorylation sites were localized in the proline-rich and C-terminal domains. However, no phosphorylation sites were detected within the two hexapeptides, 275VQIINK280 and 306VQIVYK311, that form the core of tau paired helical filaments (PHFs). Among all the detected phosphorylation sites, S202/T205/S208 within the proline-rich region (PRR) present in the phosphorylated tau protein, has been reported to induce tau self-aggregation without any exogenous aggregation inducer [28]. Phosphorylation sites near the C-terminus also preferentially promote tau self-aggregation. For instance, pseudo-phosphorylated S396/S404, recognized by the PHF-1 antibody, has been shown to increase tau aggregation in the presence of metal ion inducer [29]. The monoclonal antibody AT180 that specifically recognizes tau phosphorylation at the T231 site is currently used to define an Alzheimer’s disease (AD)-related pathological form of the phosphorylated tau protein [22]. Taken together with our determination of phosphorylated side chains, we thus expect the *E. coli*-produced phospho-0N4R tau protein to be aggregation-prone and form pathological PHF-1 and AT180-positive tau states.

#### 2.3.2. Phosphorylation of Tau Promotes Tau Cellular Uptake and Subsequent Intracellular Aggregation

**Figure 4:**
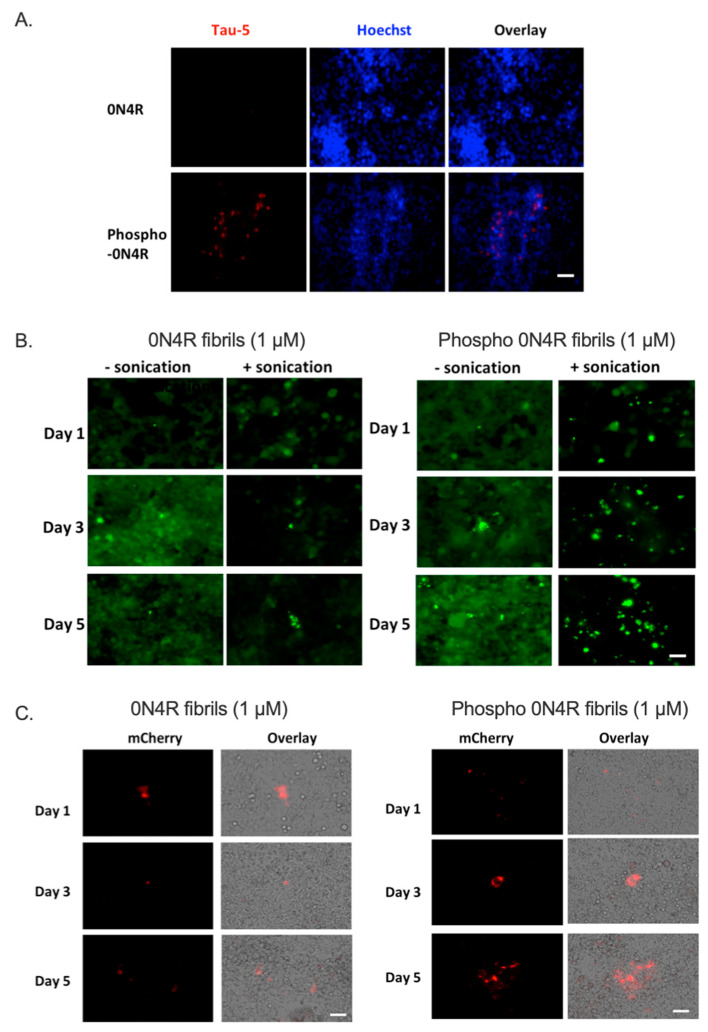
Phosphorylation of tau promotes the uptake of recombinant tau protein and the seeding propensity of tau fibrils in tau biosensor cells. (**A**) Representative images of Tau-5 immunofluorescence (red) in CHO-K1 cells after being exposed to 1 μM of monomeric 0N4R tau (0N4R) or phosphorylated tau (phospho-0N4R) for 24 h. (**B**) Fluorescence microscopy images of FRET tau biosensor cells treated for 5 days with 1 μM of tau fibrils from wild type recombinant 0N4R (0N4R) or phosphorylated 0N4R tau (Phospho-0N4R) as indicated, with (+sonication) and without sonication (−sonication). (**C**) Representative fluorescence microscopy images of tau-tau sfCherry biosensor cells treated for 5 days with 1 μM of tau fibrils from wild type recombinant 0N4R (0N4R) or phosphorylated 0N4R tau (Phospho-0N4R). Red color indicates fluorescence of reconstructed mCherry protein from tau-tau association. The scale bar denotes 100 μm.

The relevance of tau phosphorylation for cellular uptake was evaluated in mouse primary neurons in a previous study [15] by the addition of brain extracts from tau-transgenic mice models to the neuron culture. Extracts from rTg4510 mice, which overexpress human P301L tau, showed more human tau immunoreactivity as well as higher tau phosphorylation than that of rTg21221 mice, which overexpress wild type human tau. This data is consistent with a model where increased phosphorylation correlates with disease. Here, to explicitly correlate the tau protein characteristics themselves with changes to cells, and in particular the involvement of phosphorylation in tau uptake, we incubated CHO-K1 cells with purified recombinant wild type tau protein (0N4R), and phosphorylated 0N4R (referred to subsequently as phospho-0N4R). Tau uptake was visualized by immunofluorescence detection using Tau-5 antibody, after 24 h. A higher level of Tau-5+ cell fluorescence was observed for CHO-K1 cells with phospho-0N4R compared to wild type tau, suggesting the important role of phosphorylation in regulating tau uptake (Figure 4A).

We then evaluated the seeding activities of tau fibrils generated from recombinant 0N4R and phospho-0N4R tau, using previously described FRET tau biosensor cells [30], which have been used widely for assessing the seeding activities from different origins, such as tau fibrils, brain extracts from human AD patients, or tau-transgenic mouse models [31,32]. Here, the FRET biosensor cells were transduced with 1 μM of tau fibrils, induced by the anionic aggregation inducer, heparin, as described in Methods. Fluorescence microscopy was used to monitor and visualize the formation of seed-induced tau aggregation over five days (Figure 4B). Interestingly, cells treated with phospho-0N4R had a higher level of tau in the aggregated state, displaying puncta-like intracellular inclusions as early as day one. More importantly, this effect was further enhanced by one additional step of sonication, indicating both the size and form of seeds significantly affected their potential in inducing tau intracellular aggregation.

Because of the high background signal in the FRET biosensor cells, an alternative tau aggregation reporter cell line was generated by transfecting CHO-K1 cells with a novel construct—pcDNAK3.1(+)-K18-sfCherry3C_11_-2a-K18-sfCherry3C_1–10_—where K18 (the microtubule binding core domain of tau) was covalently expressed with the split fluorescent Cherry3 protein under control of the CMV promoter. The design includes the 2A self-cleaving peptide that induces ribosomal skipping during translation of the construct and generates equal ratios of two sfCherry fragments, K18-sfCherry3C_11_ and K18-sfCherryC_1–10_. Cells emit red fluorescence when the Cherry3 fragments assemble; the split fluorescence reporter has previously been used to increase signal and reduce background fluorescence from FRET protein spectral overlap [25]. Here, a stable cell line (referred to as tau-tau sfCherry biosensor) was created following two weeks of cell pool selection and one week of cell clone selection with 600 µg/mL G418 antibiotic. Using the tau-tau sfCherry cells, fluorescence was only detected following addition of exogenous tau seeds (Figure 4C). As shown in Supplementary Figure S2A, tau-tau sfCherry cells treated with empty liposome are negative for mCherry fluorescence, while tau fibrils generated from K18 tau induced intracellular tau aggregation and, therefore, showed a time-dependent increase in the mCherry signal (Supplementary Figure S2B). To evaluate the relevance of phosphorylation to the seeding activity, tau-tau sfCherry biosensor cells were transduced with 1 µM recombinant tau fibrils generated from 0N4R or phospho-0N4R. Consistently, tau-tau sfCherry biosensor cells treated with phosphorylated tau fibrils showed a higher level of mCherry fluorescence, which increased at longer incubation times (Figure 4C). Interestingly, aggregation-prone K18 fibrils had induced much more intense sfCherry fluorescence—inferring higher aggregation levels—in tau-tau sfCherry biosensor cells than the phospho-0N4R (Supplementary Figure S2B), consistent with a protective role of the N and C-terminal regions, as described previously [33]. Taken together, these data suggest that tau phosphorylation plays an important role in tau cellular uptake and subsequent intracellular aggregation. 

### 2.4. Tau Inter-Neuronal Propagation Is Promoted by Phosphorylation

Next, we established tau transcellular models with the purpose of uncovering the role of tau phosphorylation in transcellular propagation. Directly mixing tau-expressing HEK293 cells with tau FRET biosensor cells yielded no visible tau aggregates (Supplementary Figure S3A), suggesting direct contact in non-neuronal cells was not sufficient for tau transfer. An alternative approach was to use tau-containing extracellular vesicles (EVs) isolated from HEK293 cells that were applied to tau FRET biosensor cells (Supplementary Figure S4A). Although exosome delivery was confirmed by immunodetection using Tau-5 antibodies in tau FRET biosensor cells (Supplementary Figure S4B), no significant difference in overall Tau-5 signal was observed with different tau formats, possibly due to the low signal.

**Figure 5:**
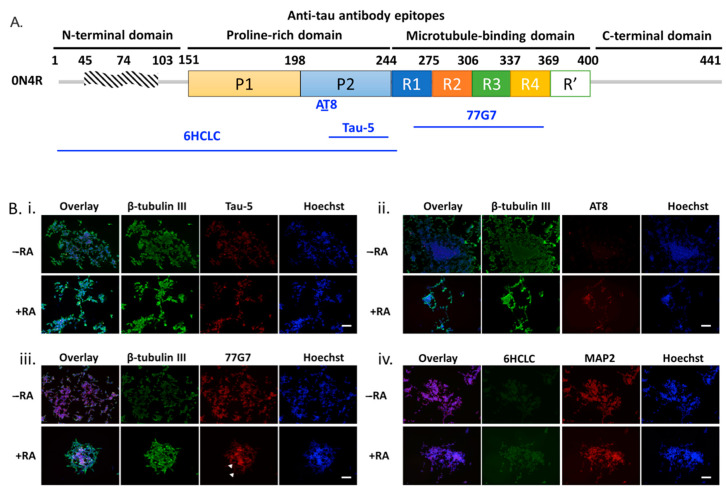
Tau phospho-isoform expression in differentiated SH-SY5Y cells. (**A**) Schematic illustration of the epitopes on 0N4R tau protein used to identify the tau expression landscape in SH-SY5Y cells. Antibodies include AT8: pSer202 and pThr205; Tau-5: aa 210–241; 77G7: 270–375; 6HCLC: aa 1–254. The residues are numbered based on the full-length tau isoform, 2N4R, with regions of interest as noted. (**B**) Representative fluorescent images of neuronal and tau markers, including β-tubulin III (Green)/Tau-5 (Red)/Hoechst (Blue) (**i**), β-tubulin III (Green)/AT8 (Red)/Hoechst (Blue) (**ii**), β-tubulin III (Green)/77G7(Red)/Hoechst (Blue) (**iii**), and 6HCLC (Green)/MAP2(Red)/Hoechst (Blue) (**iv**) for SH-SY5Y cells after 14 days of RA-induced differentiation. Scale bar = 100 µm.

To provide a more physiologically relevant cell model for investigating tau-related pathology in AD and related tauopathies, we then employed differentiated SH-SY5Y cells as recipient cells. As described in previous literature [34], SH-SY5Y-differentiated neurons contain higher levels of axonal-localized overall tau expression of all six brain-specific human tau isoforms, as well as a human brain-like tau phosphorylation pattern. For inducing neuronal differentiation, SH-SY5Y cells were treated with retinoic acid (RA) at 10 μM for 14 days, before characterization by immunostaining with different anti-tau antibodies, including Tau-5, AT8, 77G7, and 6HCLC (Figure 5A). Elongated axons were observed following RA treatment for 14 days, as visualized by immunostaining against β-tubulin III (Figure 5B). RA treatment also elevated the total tau expression in both unphosphorylated and phosphorylated forms, as indicated by the higher level of Tau-5 immunostaining, the total tau antibody detected in the +RA group compared to undifferentiated cells (-RA) (Figure 5B(i)). Additionally, higher levels of β-tubulin III and Tau-5 positive signals induced by RA treatment were also confirmed by flow cytometry (Supplementary Figure S5).

AT8, a tau antibody targeting phosphorylated Ser202/Thr205, was used to quantify the phosphorylation level of tau in differentiated and undifferentiated neurons. Not surprisingly, differentiated SH-SY5Y cells showed higher levels of tau phosphorylation, based on a higher level of AT8+ cells in the +RA group (Figure 5B(ii)). Furthermore, both groups showed significant amounts of 77G7+ cells (Figure 5B(iii)), indicating the presence of 4R tau isoforms. Interestingly, the RA group showed an increase in puncta-like structures (indicated by arrows) compared to the control (-RA) group, suggesting RA treatment may induce tau aggregation, which results from higher total tau expression as well as a higher level of tau phosphorylation. We also examined levels of the 6HCLC epitope (Figure 5B(iv)); tau 6HCLC antibody targets a large portion of the N-terminus (aa 1–254) with the capability of detecting tau isoforms with various N inserts, 0N, 1N, or 2N. Despite both RA treated and untreated cells showing the presence of 6HCLC+ neuronal cells, the RA+ group showed a much higher intensity of the 6HCLC+ cell population, suggesting a different landscape of tau isoforms (0N, 1N, or 2N) existed in the two different conditions. Together, our immunostaining data suggests that differentiated SH-SY5Y cells expressed all tau isoforms and showed phosphorylation patterns consistent with that in the human brain, making them an appropriate neuronal cell model for investigating tau pathology. 

**Figure 6:**
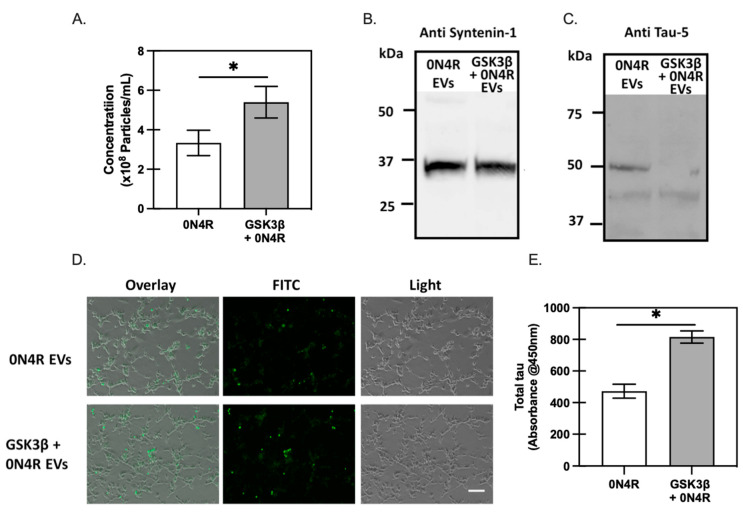
Tau inter-neuronal propagation is promoted by phosphorylation. Characterization of EVs isolated from HEK293TN cells transfected with the XPack™-0N4R construct (0N4R) without or with GSK3β (GSK3β + 0N4R) co-transfection. (**A**) ZetaView was used for measuring particle concentration (×10^8^ particles/mL). The presence of exosomes was confirmed by immunoblotting isolated exosome lysates against the exosome biosynthesis marker, Syntenin-1 with a molecular weight of 34KDa (**B**). Immunoblotting exosome lysates against tau specific antibody Tau-5 (**C**) confirmed that tau protein was packaged into exosomes. (**D**) Mature neurons differentiated from SH-SY5Y cells were imaged under a fluorescent microscope after being treated with SYTO^®^ RNASelect™ Green Fluorescent Cell Stain (ThermoFisher, Waltham, MA, USA) labeled exosomes for 24 h at 37 °C. The scale bar denotes 100 μm. (**E**) Total human tau ELISA of recipient cell lysates shows more tau was taken up from EVs isolated from GSK3β co-transfection (* represents *p* < 0.05 between two different groups, *n* = 2).

To create tau-enriched EVs, we transfected HEK293 cells with XPack™0N4R, with or without a GSK3β expression plasmid, for expressing tau packed directly into EVs (XPack™ (System Biosciences, Mountain View, CA, USA) construction in Materials and Methods). The supernatants from HEK293 cells transfected with the XPack™-0N4R construct were collected, and EVs were isolated for characterization by ZetaView (Particle Metrix, Ammersee, Germany). Co-transfecting HEK293 cells with GSK3β showed statistically equivalent sizes of isolated EVs from 0N4R control vs. 0N4R + GSK3β group (134.2 ± 2.6 nm vs. 133.7 ± 0.7 nm, respectively) based on three independent replicates. However, the GSK3β-coexpressing group had a higher particle concentration than the 0N4R group (3.3 × 10^8^ per mL vs. 5.4 × 10^8^ per mL), suggesting the presence of GSK3β facilitated the packaging of tau into EVs (Figure 6A). To confirm the identity of exosomes, isolated samples were analyzed by Western blot analysis using anti-Syntenin-1 (Figure 6B), which is a marker of the syndecan–syntenin–alix-dependent pathway of exosome biogenesis [35]. The presence of a positive band at 34 kDa for isolated EV samples confirmed the identity of exosome populations. Lastly, the presence of tau packed into the exosomes was confirmed by Western blotting using Tau-5 antibody for protein samples extracted from exosomes (Figure 6C). Note that lower levels of tau were observed from GSK3β-expressing samples; however, more particles per supernatant volume were also determined in this case, suggesting that tau protein was underrepresented in individual exosomes, as syntenin-1 levels were similar.

The uptake of fluorescently labeled exosomes by differentiated SH-SY5Y cells was analyzed by a fluorescence microscope after 24 h (Figure 6D). Here, the use of the neuronal-like recipient SH-SY5Y cells yielded many puncta like structures compared to HEK293 recipient cells (Supplementary Figure S5). Interestingly, SH-SY5Y recipient cells treated with exosomal tau phosphorylated by GSK3β had increased exosomal tau uptake, as well as higher amounts of tau aggregates (Figure 6D), which suggests a higher seeding potency of phosphorylated tau than wild type tau-containing exosomes. The transfer efficiency of tau from exosomes to neurons was examined further by measuring the total human tau level present in recipient cell lysates, via ELISA. Total tau protein present in the cell lysates of SH-SY5Y neurons treated with GSK3β-phosphorylated tau-containing exosomes was much higher than that in cell lysates from those treated with wild type 0N4R-containing exosomes (Figure 6E).

### 2.5. Phosphorylation at Ser262 Plays a Critical Role in Tau Accumulation and Toxicity

#### 2.5.1. Ser262 Pseudo-Phosphorylation Promotes Tau Secretion

**Figure 7:**
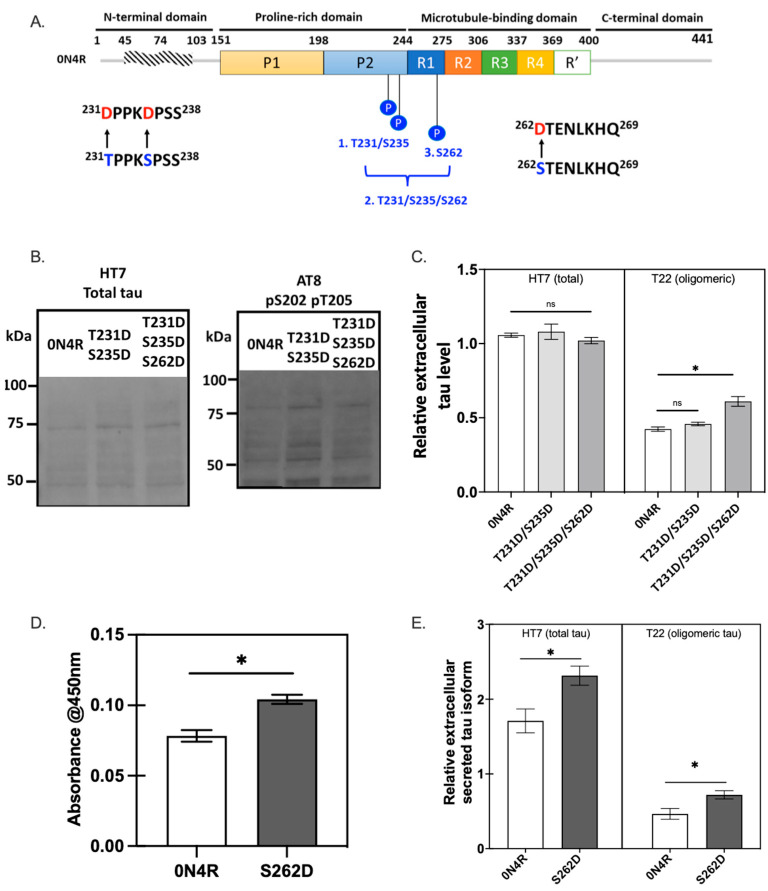
S262 phosphorylation increases tau secretion. (**A**) Schematic illustration of tau variants created via site-directed mutagenesis of wild type 0N4R construct by substituting serine at multiple phosphorylation sites (blue circles with “P” as indicated) into aspartic acid: (1) T231D/S235D; (2) T231D/S235D/S262D; (3) S262D. The residues are numbered in blue based on the full-length tau isoform, 2N4R, with regions of interest as noted by colored boxes. The amino acid sequences around these sites are also included using one letter abbreviation and the wild-type amino acid in blue, the change to aspartic indicated in red. P1 and P2 represent the proline-rich domains; R1–R4 and R’ indicated microtubule binding repeat domains. (**B**) Cell lysates from SH-SY5Y cells transfected with 0N4R tau wild-type (0N4R), T231D/S235D, or T231D/S235D/S262D, were analyzed by immunoblotting against the total tau antibody, HT7 and the phosphorylated tau antibody, AT8. (**C**) ELISA assay was used to measure the total tau, HT7, and oligomeric tau, T22, present in the conditioned medium collected from these three groups, three days following transient transfection in SH-SY5Y cells. 0N4R tau (0N4R; unshaded), T231D/S235D (light gray), or T231D/S235D/S262D (dark gray) (* represents *p* < 0.05 for 0N4R compared with S262D tau; *n* = 3) (**D**). Oligomeric tau was measured by sandwich ELISA using T22 and HT7 from the cell lysates of SH-SY5Y cells transfected with wild type 0N4R tau (unshaded) or the S262D variant (dark gray) (* represents *p* < 0.05 for 0N4R compared with S262D tau; *n* = 3). (**E**) The total tau and oligomeric tau in the conditioned medium were measured by indirect ELISA using HT7 or T22 to investigate the effects of Ser262 phosphorylation alone on tau secretion. 0N4R tau (unshaded); S262D variant (dark gray) (* represents *p* < 0.05 for 0N4R compared with S262D; *n* = 3; ns = not significant).

MARKs are non-SP/TP kinases, phosphorylating KXGS motifs in the repeat domain of tau located in the microtubule-binding region such as Ser262 and Ser356 [18], an early stage in tau phosphorylation. We created tau variants with serine or threonine residues mutated to aspartic acid at multiple phosphorylation sites that are reported to regulate tau-microtubule interaction, T231D/S235D and T231D/S235D/S262D, to mimic tau phosphorylation at these sites (Figure 7A). SH-SY5Y cells were transfected with different tau variants and incubated for three days before Western blot analysis and ELISA assay of cell lysates and culture supernatants. Double or triple pseudo-phosphorylation led to an increased level of both total and phosphorylated tau in cell lysates, as determined by Western analysis (Figure 7B), suggesting site-specific phosphorylation of tau induced tau accumulation. In contrast, a minimal effect on total tau secretion relative to wild type 0N4R was observed, although the triple pseudo-phosphorylation variant (S262D-containing) was found to promote oligomeric tau secretion to higher levels than 0N4R or T231D/S235D tau variant in SH-SY5Y cells (Figure 7C). This suggests that S262 might have particular importance in oligomer formation following phosphorylation.

To further evaluate the role of Ser262 pseudo-phosphorylation alone in tau secretion and intracellular tau aggregation, the cell lysates and culture supernatants were compared for SH-SY5Y cell cultures transfected with wild type tau or the S262D tau variant (Figure 7D,E). Similar to the results for the triple pseudo-phosphorylation variant, the S262D transfected group showed higher levels of tau oligomers in cell lysates compared to wild type 0N4R tau, based on sandwich ELISA measurements (Figure 7D). Extracellular secretion of total and oligomeric tau (Figure 7E) was also higher in S262D tau compared to wild-type 0N4R, perhaps as a result of a reduced affinity of the S262D tau variant for microtubules described in prior reports [18], leading to higher levels of free cellular tau.

#### 2.5.2. Ser262 Pseudo-Phosphorylation Alters Tau Cellular Distribution and Disrupts Microtubule Integrity

**Figure 8:**
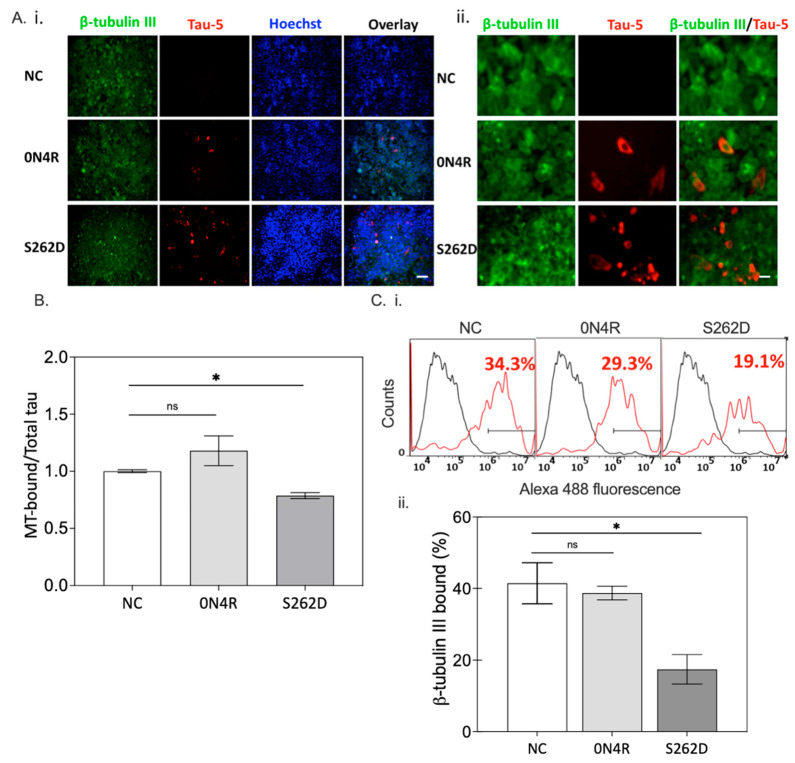
Ser262 phosphorylation alters tau cellular distribution and disrupts microtubule integrity. (**A**(**i**)) Representative fluorescent images of the neuronal marker, β-tubulin III (Green), and tau marker, Tau-5 (Red), expressed by CHO cells transiently transfected with wild-type 0N4R and S262D variant three days following transfection. Negative control (NC): CHO cells transfected with pCEP4 plasmid backbone. Scale bar = 100 µm. (**ii**). Co-staining of β-tubulin III and Tau-5 showed an overlap in cellular distribution decreased in CHO cells transfected with the S262D variant. Scale bar = 25 µm. (**B**) The amount of tau in the MT-bound fraction was normalized to total tau to get the relative tau binding affinity to microtubules for CHO cell lysates (* represents *p* < 0.05 between two different groups, *n* = 3; error bars represent SD; ns = not significant). (**C**) Cell lysates of CHO cells transfected with different constructs were processed for the in vitro microtubule binding assay (See materials and methods section). (**i**). Representative histograms of β-tubulin III by lysates of CHO cells transfected with different constructs, via flow cytometry. Black line: negative control; red line: Alexa 488 bound β-tubulin III + cell population. NC, CHO cells transfected with pCEP4 backbone. (**ii**). Quantification of β-tubulin III for all three groups 3 days post-transfection (* represents *p* < 0.05 between mean of different groups, *n* = 3; error bars represent SD; ns = not significant).

As higher levels of tau were observed for S262D tau transfection relative to wild type, the microtubule binding integrity was investigated by transiently transfecting CHO cells with constructs for the wild type 0N4R or the S262D tau variant. Here, CHO cells were used to provide greater quantities of cell lysates that were necessary for the assay. The expression of microtubule marker β-tubulin III was observed for all the groups (Figure 8A(i),(ii)), while Tau-5 was expressed only in the CHO cells transfected with 0N4R or its variant, S262D. Cells expressing the S262D variant exhibited a higher density of Tau-5 reactivity than that of the 0N4R wild type group, implying a critical role for S262 phosphorylation in tau accumulation.

The effect of Ser262 pseudo-phosphorylation on tau accumulation was also confirmed in SH-SY5Y cells (Supplementary Figure S6A,B), with the S262D group showing a higher density of Tau-5 reactivity than cells transfected with wild type 0N4R. Additionally, most β-tubulin localized to the periphery of the soma in the wild type 0N4R group, and immunostaining of it overlapped extensively with that of Tau-5. In contrast, Tau-5 in the S262D group showed differential cellular distribution, diffusing from the periphery of the cell nucleus to the cytosol, displaying a spherical inclusion in the soma next to the nucleus. In addition, there was no detectable overlap with β-tubulin, suggesting the S262D variant had decreased microtubule affinity. The affinity between tau and microtubules was then examined using an in vitro microtubule binding assay (Figure 8B). Cells expressing wild type 0N4R tau had a higher-level of microtubule-bound tau than cells expressing S262D tau, suggesting Ser262 phosphorylation reduces the affinity between tau and microtubules. This observation is consistent with morphological abnormalities of microtubules (seen via β-tubulin immunofluorescence) detected upon expression of the S262D tau variant, but not wild type tau in both cell lines (Figure 8A(ii) and Supplementary Figure S6C). A reduced level of β-tubulin was detected in S262D transfected cells compared to wild type 0N4R transfected cells (29.3% vs. 19.1%), as determined by flow cytometry (Figure 8C). Taken together, these observations suggest that phosphorylation of S262 leads to the loss of microtubule integrity and extensive tau accumulation that likely leads to eventual cell death.

### 2.6. The Interplay between Ser262 Phosphorylation and GSK3β Activity

#### 2.6.1. Temporal Relationship between Ser262 Phosphorylation and Other Phosphorylation Sites in Tau

**Figure 9:**
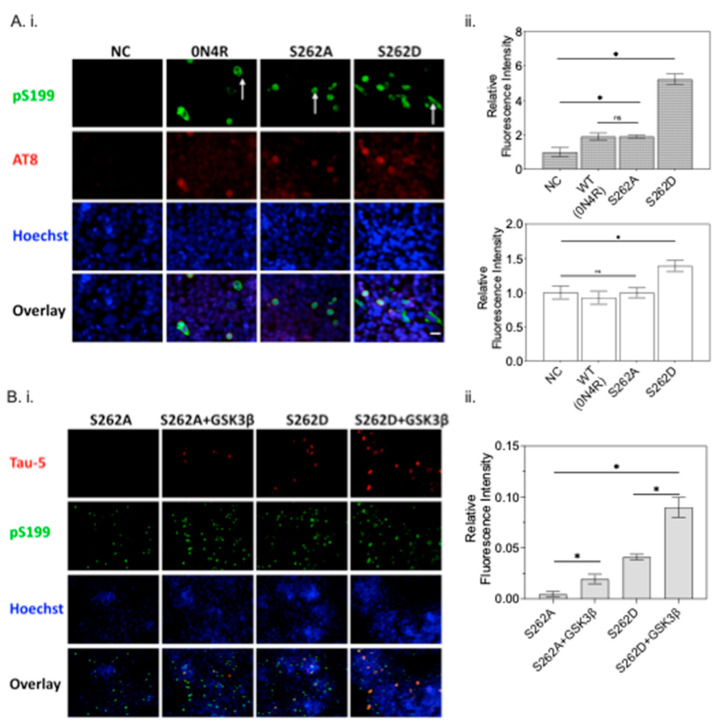
The interplay between Ser262 phosphorylation and GSK3β activity. (**A**(**i**)) Expression of phosphorylated tau at Ser199 (pS199, Green) and Ser202/Thr204 (AT8, Red) in CHO cells transfected with wild type 0N4R tau, S262A variant, or S262D variant. NC: CHO cells only. Scale bar = 25 µm. Arrows indicate the differential cellular localization of pS199 expressed by transfected cells. (**A**(**ii**)) The intensity of pS199 and AT8 immunostaining in CHO cells transfected with different constructs were normalized by Hoechst staining levels with NC set arbitrarily at 1. (* represents *p* < 0.05 between two different groups, *n* = 3; ns = not significant). (**B**) The effect of Ser262 phosphorylation on the activity of GSK3β was further examined by co-transfecting tau and GSK3β plasmids. The expression of total tau (Tau-5, Red) is shown in (**B**(**i**)). (Scale bar = 25 µm.) and quantified (**B**(**ii**)). for comparison between different groups (* represents *p* < 0.05 between two different groups, *n* = 3; ns = not significant).

To understand the factors contributing to tau accumulation by Ser262 phosphorylation, we evaluated the abnormal forms of tau by using immunohistochemistry with an AT8 antibody, which recognizes the phosphorylated Ser202 and Thr205 epitopes, and a phospho-Ser199 antibody (Figure 9A(i)) [12,36]. We found that cells transfected with S262D exhibited higher intensities of AT8+ cells and pS199+ cells than cells transfected with wild type 0N4R (Figure 9A(ii)), while non-transfected CHO cells (negative control, NC) showed negligible AT8 and pS199 immunoreactivities. Blocking the phosphorylation of Ser262 by substituting serine with alanine diminished the observed abnormal tau phosphorylation. Our results are consistent with a previous report using a *D. melanogaster* model of tauopathy, demonstrating the activity of PAR-1 as a prerequisite for the activity of GSK3β [21]. The concurrency of tau accumulation and increased pathological tau forms, AT8- and pS199-reactive, in the S262D group indicates that abnormal tau phosphorylation may be the primary cause of tau accumulation.

#### 2.6.2. The Effect of Ser262 Phosphorylation on GSK3β Activity

As the epitopes of AT8 and pS199 are the primary targets of GSK3β for tau protein [37], Ser262 phosphorylation changes to the GSK3β activity leading to increased levels of tau phosphorylation were examined. To test the effect of pseudo-phosphorylation of Ser262 on subsequent phosphorylation states, we constructed two different tau variants by substituting serine at 262 with either alanine (A) or aspartic acid (D); with the former substitution serving as a negative (non-phosphorylated) control. CHO cells were transfected with either of the tau variants along with GSK3β (Figure 9B(i)); co-transfection of Ser262D with GSK3β further enhanced tau accumulation, showing the highest Tau-5+ immunoreactivity among all the conditions tested, approximately two-fold higher than the Ser262D only group (Figure 9B(ii)). However, no major differences were observed between S262A and S262A co-transfected with GSK3β. These data suggest that Ser262 phosphorylation may precede tau phosphorylation by activating GSK3β, perhaps by exposing sites of the tau protein. 

**Figure 10:**
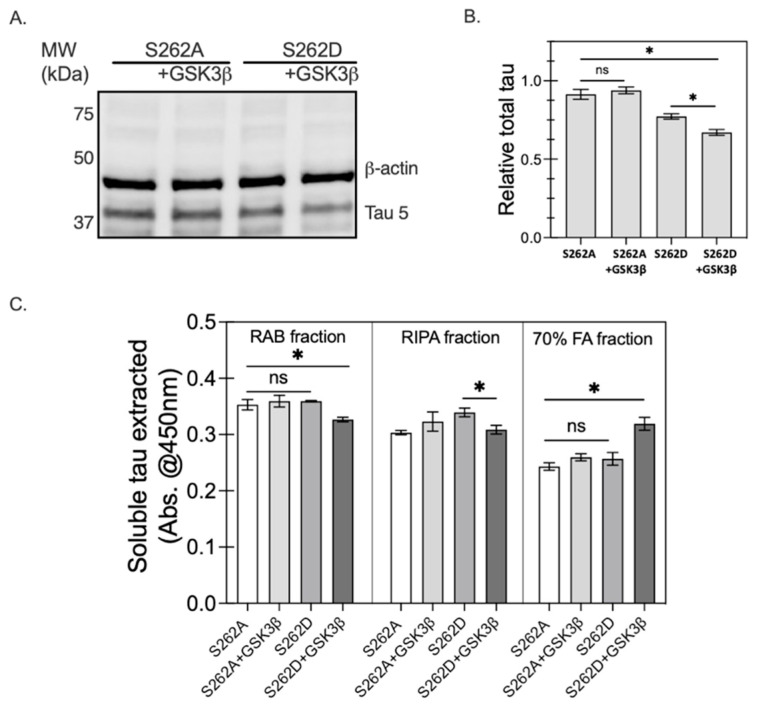
Insoluble tau levels are highest for cells co-expressing S262D and GSK3β. (**A**) CHO cells transfected with the tau S262A or S262D variant with or without co-transfection of GSK3β were lysed and the soluble portion of cell lysates were immunoblotted with anti-total tau antibody, Tau-5, and endogenous control protein, β-actin, and a representative Western is shown. (**B**) The ratio of Tau-5 to β-actin was analyzed for the different cell lysates. Total protein loaded was equivalent, though similar β-actin levels were present in each sample. Relative soluble tau was determined by quantifying Tau5+ band intensity relative to β-actin to quantify the soluble tau content (* represents *p* < 0.05 between two different means, *n* = 3, with error shown as the SEM). (**C**) Crude cell lysates from different groups were sequentially extracted by RAB, RIPA, and 70% FA, as indicated, and evaluated by total tau ELISA assay. A significant decrease was observed in soluble tau levels in RAB and RIPA fractions and an increase in the 70% FA fraction for cell lysates from S262D with GSK3β co-expression as compared to the other three cell lysates. Cell lysates for tau S262A without (unshaded) or with (light gray) co-transfection of GSK3β or S262D variant without (medium gray) or with (dark gray) co-transfection of GSK3β (* represents *p* < 0.05 between means, *n* = 3, with error shown as the SEM; ns = not significant).

The levels of soluble tau in the cell lysates were evaluated by Western blot analysis. We found that S262D co-transfected with GSK3β exhibited the lowest level of detergent soluble tau among all the groups tested (Figure 10A,B), suggesting the formation of insoluble tau increased. To test this likelihood, we conducted a sequential protein extraction of total cell lysates, followed by ELISA assay to evaluate the detergent-soluble fraction from the total cell lysates recovered under each condition using established methods, where reassembly (RAB) buffer is the mildest and 70% formic acid the strongest [38]. Consistently, cell lysates from cell co-expressing S262D with GSK3β also showed the lowest RAB and RIPA soluble tau among all the groups (Figure 10C). Consistent with the low detergent soluble levels of tau, lysates of cells expressing S262D with GSK3β exhibited the highest amount of 70% formic acid (FA)-soluble tau (Figure 10C). These results have confirmed our hypothesis that Ser262 pre-phosphorylation promotes GSK3β-driven tau phosphorylation, tau accumulation, and insoluble tau formation.

#### 2.6.3. The Role of Ser262 Phosphorylation on β-Amyloid-Induced Tau Pathology

**Figure 11:**
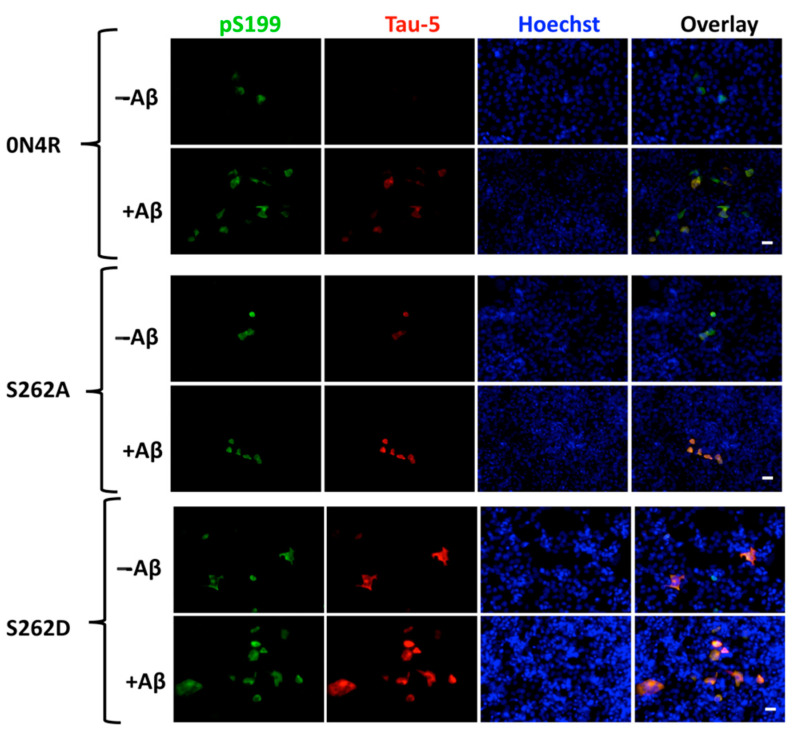
The presence of Ser262 phosphorylation exacerbates β-amyloid-induced tau pathology. CHO cells transfected with wild type 0N4R tau, S262A, or the S262D variant were untreated (−Aβ) or treated with 0.5 μM of Aβ (+Aβ) for 72 h. prior to analysis. Cells were replated into 24-well plate for performing immunostaining using tau antibodies, pS199 (Green), Tau-5 (Red), and nuclear Hoechst (Blue) staining, as indicated, to visualize the combinational effects of S262 phosphorylation and Aβ on tau accumulation and phosphorylation. Scale bar = 100 μm.

Accumulating evidence suggests that tau acts as the mediator between amyloid-beta (Aβ) and subsequent neurotoxicity, including impaired synaptic plasticity and tau inclusions [39,40]. One previous study using a *D. melanogaster* model demonstrated that amyloid beta-driven dendritic spine defects and synapse loss were mediated by Ser262 phosphorylation [39]. Here, we evaluated the role of Ser262 phosphorylation in Aβ-induced tau accumulation in CHO cells (Figure 11). A significant increase in the intensity of pS199+ and Tau-5+ cells was observed in the 0N4R group 72 h after the addition of amyloid-beta. However, by blocking the phosphorylation of Ser262 via the S262A substitution, amyloid beta addition showed minimal pS199+ and Tau-5+ cells. Despite the higher level of tau accumulation in the S262D group, addition of amyloid-beta exacerbated pS199+ and Tau-5+ immunoreactive cells detected. Our results suggest Ser262 phosphorylation is a necessary step in amyloid beta-induced tau accumulation and phosphorylation. 

## 3. Discussion

### 3.1. GSK3β-Induced Tau Hyperphosphorylation Promotes the Spread of Tau Pathology

Tauopathies, including Alzheimer’s Disease (AD), are characterized by the formation of toxic NFTs [1], which are comprised of aggregates of abnormally phosphorylated tau protein. Tau phosphorylation by GSK3β is associated with multiple neuropathological events during the progression of AD [37], including the formation of NFTs [40], the formation of oligomeric Aβ in neurons, and even dendritic spine loss induced by Aβ [41]. GSK3β attenuation in a tau transgenic mouse model was shown to inhibit tau hyperphosphorylation, reduce the formation of tangle-like aggregates of tau in hippocampal neurons, and alleviate amyloid-β induced neurotoxicity [42,43]. The toxic tau species, which can induce intracellular aggregation and be internalized by adjacent cells, were previously identified as phosphorylated tau in the soluble form with a relatively higher molecular weight [15]. Dephosphorylation of tau oligomers isolated from the AD brain attenuated the capture of tau monomers and templating further tau aggregation [44]. Although those studies revealed a possible relationship between tau phosphorylation and tau pathology, our study has identified and associated abnormal tau phosphorylation, oligomerization, release, and uptake with GSK3β activity, furthering our mechanistic understanding of tau pathology. 

The effects of GSK3β-related tau phosphorylation on each step of transcellular tau propagation, including tau release, uptake, and intracellular aggregation, were examined independently in our studies. Here, overexpression of GSK3β in SH-SY5Y neuroblastoma cells increased not only the levels of tau phosphorylation (Figure 2A,B), but also enhanced the cellular immunoreactivity to the tau oligomeric antibodies T22 (Figure 2A–C). Additionally, uptake of recombinant wild type full-length human tau in the form of monomers or fibrils was enhanced by GSK3β-driven phosphorylation (Figure 4B), suggesting tau phosphorylation facilitated cellular tau uptake (Figure 4A). GSK3β-induced phosphorylation led to a higher level of tau seeding activity in FRET biosensor cells treated with tau fibrils generated from recombinant phosphorylated 0N4R. Our study also tested the roles of sites phosphorylated by GSK3β (Figure 3E and Figure 4) that have been associated with AD brain-relevant tau. 

Further, we established an in vitro tau transcellular model by treating differentiated SH-SY5Y cells with phosphorylated tau-containing exosomes (Figure 6). The crucial role of exosomes as transcellular vehicles in the spread of pathological tau has been widely described in the field of tauopathies [45]. Exosome-associated tau derived from AD brains were more efficient in inducing endogenous templated tau misfolding as compared to multiple species of physiological levels of free tau. Further, exosomal tau derived from AD brain were more transmissible than exosomal tau derived from non-demented controls, where injection into aged mouse brain led to abnormally phosphorylated tau accumulated in glutamic acid decarboxylase 67 (GAD67) GABAergic interneurons and inhibited postsynaptic currents, suggesting the cell type-specific susceptibility of tau pathology in the progression of tau pathology [45]. Here, substantial effects on tau aggregation were observed upon exosomal tau addition to retinoic-acid differentiated SH-SY5Y cells, demonstrating a possible neuronal subtype-specific tau vulnerability. Additionally, the transmissivity and toxicity of tau species was greatly enhanced by phosphorylation, as evident by a marked increase in tau aggregates formed in SH-SY5Y cells treated with exosomal tau pre-phosphorylated by GSK3β (Figure 6).

### 3.2. Ser262 Phosphorylation Induces Abnormal Tau Phosphorylation and Tau Accumulation

Among the possible tau phosphorylation sites, Ser262 has accumulated focus because it is localized in the KXGS motif of the first repeat domain in the microtubule-binding region of tau. Previous evidence showed that MARKs, but not proline-directed tau kinases MAPK and GSK3β, are responsible for phosphorylating sites within microtubule-binding regions of tau, including Ser293, Ser324, Ser356, and Ser262. Of particular interest, tau phosphorylation at Ser262 has shown prominent effects on tau toxicity [46,47]. However, multiple studies have shown that tau phosphorylation at Ser262 is not linked directly to tau aggregation or PHF formation but rather plays a role in inhibiting tau aggregation to some extent [28,31].

Here, the role of Ser262 phosphorylation in inhibiting tau-microtubule interactions was examined using a pseudo-phosphorylation tau variant, S262D. Reduced affinity between S262D tau and microtubules was compared with cells expressing wild type 0N4R tau (Figure 8). Our results are consistent with previous evidence showing phosphorylation of Ser262 strongly affects the affinity between tau and microtubules and facilitates the disassembly of tau from microtubules [48]. Moreover, the disrupted microtubule integrity caused by the S262D variant led to much lower β-tubulin+ immunoreactivity and morphological abnormalities of β-tubulin in S262D-expressing cells (Figure 8A,C), similar to previous reports [18,20]. Thus, it was surprising to observe a higher amount of tau protein inclusions for S262D tau accumulated in the cell soma, which may contribute largely to the Ser262 phosphorylation-induced tau toxicity (Figure 8A). Similar effects have been reported in a previous study using transgenic *D. melanogaster* overexpressing human MARK4, which increased the levels of total tau in vivo and enhanced tau-induced neurodegeneration [49]. Interestingly, pseudo-phosphorylation at Ser262 increased the levels of tau phosphorylated at additional sites, as recognized by AD-relevant antibodies [12] including phospho-Ser199 and AT8 (pS202 and pT205) (Figure 9). Taken together, these results suggest a possible causative relationship between abnormally phosphorylated tau and tau accumulation caused by Ser262 phosphorylation, with Ser262 phosphorylation as a prerequisite for the subsequent phosphorylation at AT8 and AT100 epitope sites [21]. 

### 3.3. GSK3β Acts as the Intermediator between Ser262 Phosphorylation and Its Subsequent Tau Accumulation

Our study has additional implications for the formation of insoluble tau aggregates or PHF. We found that using a S262A variant that blocked tau phosphorylation at Ser262 prevented GSK3β from phosphorylating tau and, therefore, led to reduced tau accumulation (Figure 9 and Figure 10). This result suggests the GSK3β activity for tau may rely strongly on the presence of Ser262 phosphorylation, rather than the proposition from previous studies that Ser262 phosphorylation inhibits insoluble tau aggregates or PHF formation [28,31,48]. In a *D. melanogaster* model of tauopathy mutating PAR-1 [21], this fly homolog of MARK kinase led to a large reduction in tau phosphorylation at multiple AD-associated sites including AT100 (pT212 and pS214) and AT8 (pS202 and pT205), consistent with our observations. The overall effects of Ser262 phosphorylation on tau pathology and the interdependency between Ser262 phosphorylation and GSK3β have suggested sequential events, where pre-phosphorylation at Ser262 activates GSK3β activity on tau, leading to increasing phosphorylation at multiple sites, leading to subsequent tau accumulation. 

Amyloid-β (Aβ), another hallmark of AD, has been previously suggested to exert its toxic effects on tau via phosphorylation at Ser262 [50]. Indeed, S262D tau showed a higher level of phosphorylation and accumulation when exposed to Aβ oligomers (Figure 11). Without access to Ser262 (i.e., in S262A tau), Aβ oligomers showed limited effects on inducing tau toxicity (Figure 11). Given the temporal relationship between GSK3β and Ser262 phosphorylation, Aβ may act through MAPK to phosphorylate Ser262, activating subsequent increased GSK3β activity and further tau phosphorylation for the progression of tau pathology. This model is consistent with a study finding that GSK3β was necessary to mediate the neurotoxicity of Aβ [43]. However, the dependency of GSK3β activation in mediating Aβ-tau toxicity needs further investigation. 

## 4. Materials and Methods

### 4.1. Cell Culture and Differentiation

SH-SY5Y neuroblastoma cells (CRL-2266™ from ATCC; Manassas, VA, USA) were cultured in a 1:1 mixture of HAMS and Minimal Essential Media, containing 10% fetal bovine serum (FBS). HEK293 cells (CRL-1573™, ATCC), Tau RD P301S FRET Biosensor cells (CRL-3275™, ATCC), and HEK293TN cells (System Biosciences, Mountain View, CA; LV900A-1) were cultured in DMEM supplemented with 10% fetal bovine serum (FBS) (Life Technologies, Carlsbad, CA, USA). CHO cells (CRL-9618™, ATCC) were cultured in HAMS (ATCC) supplemented with 10% FBS. Cell cultures were maintained in a humidified atmosphere of 5% CO_2_ at 37 °C and cultured in CELLSTAR 75 cm^2^ culture flasks with filters. For differentiation, SH-SY5Y cells were grown for 14 days in DMEM/F12 supplemented with 2% FBS containing 10 μM retinoic acid (RA, MilliporeSigma, Burlington, MA, USA; R2625).

### 4.2. Plasmid and Primers

For recombinant tau expression in *E. coli* (Rosetta™(DE3) cells (MilliporeSigma, Burlington, MA, USA; 70954)), human microtubule-associated protein tau isoform, 0N4R, was subcloned into pETM-13 (https://grp-pepcore.embl-community.io/vectors/index.html; last accessed 29 August 2022) (0N4R isoform described in Morozova et al. [51]), and GSK-3β (a gift of the Woodgett lab, RRID:Addgene_15898) was subcloned into pBAD (EMBL). Both plasmids were generated using ligation-independent cloning [52].

For mammalian expression, 0N4R cDNA sequence was PCR-amplified and subcloned into pCEP4 (ThermoFisher, Waltham, MA, USA) using the following pair of primers: 0N4R_pCEP4_NotI_fwd (TAAGCAGCGGCCGCATGGCTGAGCCCCGCCAGGATTC) and 0N4R_ pCEP4_XhoI_rev (TGCTTACTCGAGCTATCACAAACCCTGCTTGGCC). Numbering for amino acid residues in tau isoforms is presented as typical in the field, based on the full-length tau isoform, 2N4R. The tau variants, T231D/S235D and S262D were generated based on the wild type pCEP4-0N4R construct using site-directed mutagenesis (Q5^®^ Site-Directed Mutagenesis Kit, New England Biolabs, Ipswich, MA, USA), by replacing T231, S235, and S262 with aspartate (D). The primers were purchased from IDT and used for site-directed mutagenesis according to the manufacturer’s instructions. T231D/S235D was generated using the following primers (substitution from WT underlined): T231D_fwd: 5′-AGTGGTCCGTGATCCACCCAAGTCG-3′, T231D_rev: 5′-GCCACCTTCTTGGGCTCC-3′, S235D_fwd: 5′-TCCACCCAAGGATCCGTCTTCCGCC-3′, S235D_rev: 5′-TCACGGACCACTGCCACC-3′. T231D/S235D/S262D and S262D were generated based on T231D/S235D tau variant and pCEP4-0N4R construct, respectively, by replacing serine 262 to aspartate using the following primers: S262D_fwd: 5′-CAAGATCGGCGATACTGAGAACCTG-3′, S262D_rev: 5′-GACTTGACATTCTTCAGG-3′. Lastly, tau variant S262A was generated by replacing serine at position 262 to alanine (A) using the following primers: S262A_fwd: 5′-CAAGATCGGCGCTACTGAGAACC-3′, S262A_rev: 5′-GACTTGACATTCTTCAGGTC-3′. The pcDNA3 HA-GSK3β plasmid was a gift from the Woodgett lab (RRID:Addgene_14754). The 0N4R cDNA sequence was also subcloned into the exosome-based XPack™ plasmid (System Biosciences, Mountain View, CA; XPAK710PA-1) using the following pair of primers: 0N4R_XPack_XhoI_fwd (TAAGCACTCGTATGGCTGAGCCC-CGCCAGGAGTTC) and 0N4R_XPack_NotI_rev (TGCTTAGCGGCCGCTATCACAAA-CCCTGCTTGGCC) to create the XPack-0N4R construct. Variants were confirmed by DNA Sequencing.

### 4.3. Transient Transfection

SH-SY5Y neuroblastoma cells, HEK293 cells and CHO-K1 cells were transiently transfected using Lipofectamine™ 3000 transfection reagent (ThermoFisher, Waltham, MA, USA) according to the manufacturer’s instructions. Briefly, approximately 0.5 × 10^6^ cells were plated in a 6-well plate the day before transfection, in order to reach 90% confluency on the transfection day. For every reaction, 2.5 μg of plasmid was diluted in 125 µL Opti-MEM™ medium (ThermoFisher, Waltham, MA, USA) and 5 µL of P3000 reagent was added. In the second tube, 5 μL of Lipofectamine™ 3000 was diluted in 125 µL Opti-MEM™ medium. Tubes were mixed and incubated for 15 min at room temperature. At the end of the incubation, 250 µL of the complexes were added dropwise to the cells and mixed thoroughly by rocking the plate manually. The same procedure was followed for co-transfection of wild type tau or tau variants and the pcDNA3 HA-GSK3β plasmid (GSK3β), by mixing in a 1:1 ratio of the two constructs. The cells were subjected to downstream characterization 48 h after transfection.

For exosomal tau expression, 8.0 × 10^6^ HEK293TN producer cells per 150 mm cell culture plate were seeded in DMEM supplemented with 10% FBS without antibiotics at least 20 h prior to transfection. The cells were incubated at 37 °C and 5% CO_2_ to reach approximately 80% confluency the following day. The day of transfection, 10 μg of XPack-0N4R was diluted in 1.5 mL Opti-MEM TM medium (ThermoFisher, Waltham, MA, USA) containing 35 µL of P3000 reagent in the first tube. In the second tube, 41 μL of Lipofectamine™ 3000 was diluted in 1.5 mL Opti-MEM TM medium. Both tubes were mixed and incubated for 15 min at room temperature. At the end of the incubation, 3 mL of the complexes were added dropwise to the cells and mixed thoroughly by gently shaking the plate. After six hours of incubation at 37 °C and 5% CO_2_, the transfection medium was replaced with 20 mL of fresh DMEM supplemented with 10% FBS, and the cell culture was incubated overnight. The supernatant was collected into a 50-mL sterile, capped conical centrifuge tube, both 24 h and 48 h after fresh media exchange. The collected supernatants were pooled together and centrifuged at 3000× *g* for 15 min at room temperature to pellet cell debris. The supernatant was transferred into a new tube for further exosome isolation. The same procedure was followed for the co-transfection of XPack-0N4R and pcDNA3 HA-GSK3β plasmid (GSK3β), by mixing a 1:1 ratio of the two constructs.

### 4.4. Recombinant Protein Expression, Purification, and Fibril Preparation

For wild type 0N4R expression and purification, the pETM-13-0N4R tau plasmid was transformed by heat-shock into chemically competent Rosetta™(DE3) cells (MilliporeSigma, Burlington, MA, USA; 70954) using standard techniques [53], and plasmid-containing clones selected with LB containing 34 µg/mL chloramphenicol and 50 μg/mL kanamycin. For expressing phosphorylated tau, the pETM-13-0N4R tau plasmid was co-transformed with pBAD-GSK3β plasmid into Rosetta-DE3 cells and plated on LB containing 34 µg/mL chloramphenicol and 50 µg/mL kanamycin. As needed, 100 µg/mL ampicillin was added for pBAD-GSK3β plasmid selection. Individual plasmid-containing colonies were grown in LB media containing 34 µg/mL chloramphenicol and 50 µg/mL kanamycin, and with 100 µg/mL ampicillin as needed to maintain the GSK-3β plasmid. For protein production, cells were inoculated and grown in Terrific Broth (24 g/L Yeast extract, 20 g/L tryptone, 4 mL/L glycerol) at 37 °C until the optical density (OD_600_) was between 0.5 and 0.9. To induce tau expression, isopropyl β-D-1-thiogalactopyranoside (IPTG) was added to a final concentration of 0.5 mM, and the culture was grown for 4 h at 37 °C. For phospho-tau expression, the cells co-transformed with pETM-13-0N4R and pBAD-GSK3β also received arabinose at a final concentration of 0.1%, added at the same time as the IPTG. The bacterial cells were pelleted by centrifugation at 10,000× *g* and tau directly purified, or the pellet was frozen at −80 °C prior to purification. 

Tau proteins were purified as described previously [54]. Briefly, pelleted cells were resuspended in a high-salt buffer (0.1 M MES, 1 mM EGTA, 0.5 mM MgSO_4_, 0.75 M NaCl, 0.02 M NaF, 1 mM PMSF, pH 7.0), disrupted via sonication, and centrifuged at 3200× *g* to remove insoluble debris. The supernatant was decanted and then boiled for 10 min, cooled on ice for 15 min, and then centrifuged at 3200× *g* for 10 min to remove and discard precipitated protein. The supernatant was decanted and dialyzed overnight at 4 °C in column wash buffer (20 mM PIPES, 10 mM NaCl, 1 mM EGTA, 1 mM MgSO_4_, 2 mM DTT, 0.1 mM PMSF, pH 6.5). The dialyzed lysate was applied to an SP-sepharose cation-exchange column on a BioLogic DuoFlow chromatography system (Bio-Rad, Hercules, CA, USA); following application, the column was washed for 10 column volumes and protein eluted with 0.4 M NaCl. Protein-containing fractions were combined, buffer-exchanged into PBS, and concentrated using Amicon^®^. Pro purification concentrators (10 kDa MWCO, ACS501002, MilliporeSigma, Burlington, MA, USA). The protein concentration was determined by measuring the absorbance at 280 nm (A280) using Beer’s Law with ϵ = 7450 M^−1^ cm^−1^ and MW= 40 kDa. A typical final yield was 10–12 mg of tau per liter of culture. 

To induce fibrillization of tau monomer, 8 μM of wild type 0N4R or phosphorylated 0N4R tau, was incubated with 10 mM DTT for 1 h at room temperature and incubated further in 10 mM HEPES (N-2-hydroxyethylpiperazine-N′-2-ethanesulfonic acid), 100 mM NaCl, and 8 μM heparin at 37 °C for 48 h without agitation. Immediately before use, all fibrils were sonicated in the Qsonica Q700 water bath sonicator (Cole Palmer, Vernon Hills, IL) at power 65 for 30 s.

### 4.5. Amyloid β Treatment

To prepare oligomers of Aβ42 peptides, Aβ (1–42) (Bachem, Bubendorf, Switzerland) was fully dissolved at 0.5 mg/mL in hexafluor-2-propanole (HFIP, Sigma, Burlington, MA, USA). The solution was dispensed as 10 μL aliquots into siliconized Snap-Cap microtubes, placed in a desiccator to evaporate HFIP, and thereafter stored at −80 °C. Oligomer solutions were prepared freshly for each experiment as follows. The stock was dissolved in 10 µL of DMSO (to 105 μM) and incubated for 3 h at room temperature [55]. Aβ42 peptides at 0.5 μM were added to CHO-K1 cells transfected with wild type 0N4R, S262A, or S262D plasmids for 72h prior to characterization.

### 4.6. Exosome Isolation, Characterization, Labeling, and Target Cell Treatment

Exosomes were isolated from HEK293TN cells expressing XPack™ constructs using Total Exosome Isolation (Life Technologies, Carlsbad, CA, USA; 4478359) reagents following the manufacturer’s instructions. The isolated exosome pellets were resuspended in 400 μL sterilized 1× PBS buffer. The concentrated exosomes were diluted 100-fold using sterilized PBS for further biophysical characterization using ZetaView, Nanoparticle Tracking Analysis (NTA) instrument (Particle Metrix) for measuring particle size and concentration. For in vitro labeling of exosomal RNA, 1 μL of SYTO^®^ RNASelect™ Green Fluorescent Cell Stain (ThermoFisher, Waltham, MA, USA; S32703) was added to the diluted exosome samples and incubated at 37 °C for 30 min. For determining uptake of the labeled exosomes by recipient cells, 2 × 10^5^ differentiated SH-SY5Y cells were seeded into a single well of 24 well-plate and cultured overnight. Fluorescently labeled exosomes were added to the cells and incubated at 37 °C for 24 h before analyzing by fluorescence microscopy.

### 4.7. Liposome-Mediated Transduction of Tau Seeds into FRET Biosensor Cells

Tau RD P301S FRET Biosensor cells (ATCC CRL-3275™) were plated at a density of 2 × 10^5^ cells per well in a 6-well plate. Eighteen hours later, at 60% confluency, cells were transduced with tau fibrils per established methods [30]. Transduction complexes were made by combining [125 µL Opti-MEM (Gibco) + 5 µL Lipofectamine 3000 (ThermoFisher, Waltham, MA, USA)] with [125 μL Opti-MEM + 1 μM tau fibrils] for a total volume of 250 µL per well. Liposome preparations were incubated at room temperature for 20 min before addition to cells. Cells were incubated with transduction complexes for 24 h prior to media replacement, then cells were cultured for another four days. Cells were visualized and imaged every other day using fluorescence microscopy (Keyence, Cranberry Twp, PA, USA; BZ-X810).

### 4.8. Tau-Tau sfCherry Biosensor Cell Line Construction and Transduction

The complementation of split fluorescent (sf) proteins to study and quantify intracellular protein–protein interactions avoids the complicated measurement of FRET and therefore, provides an efficient way to measure and detect tau-tau interactions inside the cells [25]. We designed a DNA fragment containing sequences for three components, the fusion protein of K18 tau—the core MT-binding domain from Leu243-Glu372—and the first ten β-strands of red fluorescent protein, Cherry3, named sfCherry3_1–10_, the 2A peptide, and the fusion protein of K18 and the last β-strand of Cherry, named as, sfCherry3_11_. The DNA fragment was synthesized and subcloned into the pcDNA3.1(+) plasmid backbone via restriction enzyme cloning using BamHI and XhoI as the restriction sites (GeneArt, ThermoFisher, Waltham, MA, USA) to create a pcDNA3.1(+)-vector construct of K18-sfCherry3C_11_-2A-K18-sfCherry3C_1–10_ under the CMV promotor (i.e., pcDNA3.1(+)-CMV-K18-sfCherry3C_11_-2A-K18-sfCherry_1–10_).

CHO cells were transfected with this plasmid using Lipofectamine™ 3000 following the manufacturer’s instructions. Three days post-transfection, limited dilution single-cell cloning was performed on the cell pool to generate single-cell clones for further expansion in multiple-sized cell culture plates. The final clonal cell line stably expressing pcDNA3.1(+)-CMV-K18-sfCherry3C_11_-2A-K18-sfCherry_1–10_ is referred to as the tau-tau sfCherry biosensor. For testing tau aggregation using the newly created tau-tau sfCherry biosensor cells, cells were plated at a density of 2 × 10^5^ cells per well in a 6-well plate. Eighteen hours later, at 60% confluency, cells were transduced with fibrils generated from purified recombinant 0N4R tau monomers and phosphorylated 0N4R tau monomers (phospho-0N4R). Transduction complexes were made by combining [125 µL Opti-MEM (Gibco) +5 µL Lipofectamine 3000 (Invitrogen)] with [125 μL Opti-MEM + 1 μM tau fibrils] for a total volume of 250 µL per well. Liposome preparations were incubated at room temperature for 20 min before adding to cells. Cells were incubated with transduction complexes for 24 h. prior to media replacement and then cultured for another 4 days. Cells were visualized and imaged every other day using a fluorescent microscope (Keyence, Cranberry Twp, PA, USA; BZ-X810).

### 4.9. Protein Extraction

Cell were trypsinized and washed with ice-cold PBS prior to suspension in RAB (G-Biosciences) or RIPA lysis buffer (150 mM NaCl, 0.1% (*v*/*v*) Triton X-100, 0.5% (*w*/*v*) sodium deoxycholate, 0.1% (*w*/*v*) SDS, 50 mM Tri-HCl), and Halt™ Protease and Phosphatase Inhibitor Cocktail (ThermoFisher, Waltham, MA, USA) for protein extraction. The cell lysate was maintained at constant agitation for 30 min at 4 °C followed by centrifugation at 16,000× *g* for 20 min at 4 °C. Protein concentration was determined by BCA assay (Thermo Fisher Scientific, USA).

### 4.10. Sequential Protein Extraction

Extraction was performed as described previously with minor modifications [56]. Cell pellets were homogenized in 500 μL of RAB buffer (G-Biosciences, St. Louis, MO, USA) supplemented by Halt™ Protease and Phosphatase Inhibitor Cocktail (Thermo Fisher Scientific, Waltham, MA, USA). In brief, the samples were centrifuged at 20,000× *g* for 40 min at 4 °C. The supernatants were collected as RAB soluble fractions and pellets were resuspended in 500 μL of RIPA buffer (150 mM NaCl, 0.1% (*v*/*v*) Triton X-100, 0.5% (*w*/*v*) sodium deoxycholate, 0.1% (*w*/*v*) SDS, 50 mM Tri-HCL, and Halt™ Protease and Phosphatase Inhibitor Cocktail), centrifuged at 20,000× *g* for 40 min at 4 °C. The supernatants were collected as RIPA soluble fractions. The pellets were further resuspended in 500 μL of 70% formic acid supplemented by Halt™ Protease and Phosphatase Inhibitor Cocktail and centrifuged at 20,000× *g* for 40 min at 4 °C. The supernatants were collected as 70% formic acid fractions. Protein concentrations in the soluble fractions were measured using BCA assay. Equal volumes of the soluble fraction were analyzed by ELISA assay.

### 4.11. In Situ Microtubule Binding Assay

Three days after transient transfection, 8.0 × 10^5^ CHO cells transfected with pCEP4, pCEP4-0N4R, or pCEP4-S262D plasmids were dissociated and replated in 6 well plates. The next day, cells were trypsinized and washed with ice-cold PBS prior to suspension in 500 μL of Buffer-C+ (50 mM HEPES pH 7.1, 1 mM MgCl_2_, 1 mM guanosine-5′-triphosphate, 1 mM Dithiothreitol, 1 mM EGTA, 1% (*v*/*v*) 100× protease inhibitor cocktail (Sigma-Aldrich)). Cell lysates were centrifuged at 1000× *g* for 10 min at 4 °C, and 250 μL of the supernatant was retained (total protein). Then, 20 μM Taxol (Sigma-Aldrich) diluted in dimethyl sulfoxide (DMSO) was added to the remaining 250 μL and incubated at 37 °C for 30 min, followed by centrifugation at 100,000× *g* for 60 min at 37 °C. After centrifugation, the pellet, containing microtubule polymers, was resuspended in 500 μL of Buffer-C+ (microtubule (MT)-bound fraction). Protein concentrations of total tau and microtubule-bound fractions were measured using the BCA assay. Equal volumes of all fractions were analyzed by ELISA assay using total tau antibody, HT7, to quantity the amount of tau in different fractions. The amount of tau in the microtubule-bound fraction was normalized to total tau to obtain the relative tau binding affinity to microtubules for CHO cell lysates transfected with different tau variants.

### 4.12. SDS-PAGE and Western Blotting

Prior to gel electrophoresis, cell lysate, exosome pellets, or exosome lysate suspended in RIPA lysis buffer was mixed with a 4× Laemmli sample loading buffer (Bio-Rad, Hercules, CA, USA) and boiled at 95 °C for five mins. Then, 20 μg of protein were loaded into the wells of a Mini-PROTEAN TGX Precast Gel (Bio-Rad, USA), along with Precision Plus Protein™ WesternC™ standards (Bio-Rad, USA), and electrophoresed for 30 min at 225 V. Proteins were then transferred to a 0.2 µm preassembled PVDF membrane (Bio-Rad, USA) using the Trans-Blot Turbo Transfer System (Bio-Rad, USA). The membranes were subjected to Western analysis as described previously [57]. Briefly, membranes were incubated with 5% nonfat dried milk in 1X Tris-buffered saline containing 0.1% (*v*/*v*) Tween-20 (TBS-T) buffer for 1 h at room temperature to block non-specific binding and incubated with the primary antibodies, anti-phospho-Tau (Ser199) antibodies, (pS199, Life Technologies, 44734G), anti-phospho-Tau (Ser202, Thr205) antibody (AT8, Life Technologies, MN1020), anti-Tau (phospho T231) antibody (T231, Abcam, ab151559), anti-tau antibody (Tau-5, Life Technologies, MA512808), anti-tau antibody (HT7, Life Technologies, MN1000), anti-tau oligomeric antibody (T22, Millipore Sigma, ABN454), anti-tau (77G7, BioLegend, San Diego, CA, USA; SIG-39405), anti-tau antibody (6HCLC, Life Technologies, 710080), anti-Syntenin (Synteinin-1, Abcam, ab133267), anti-MAP2 (MAP2, Millipore Sigma, M1406) or endogenous control protein, anti-β-actin (Cell Signaling, Danvers, MA, USA; 3700T), in TBS-T containing 5% (*w*/*v*) BSA overnight at 4 °C. After washing with 1X TBS-T, the membrane was incubated with the corresponding secondary antibodies (Supplementary Table S1) for 1 h. at room temperature. The membranes were imaged with ChemiDoc MP Imaging System (Bio-Rad, USA) and the band intensities of target proteins were normalized to the endogenous control protein, β-actin (Cell Signaling, 8H10D10), using ImageJ software (Bethesda, MD, USA; https://imagej.nih.gov/ij/ last accessed on 28 August 2022).

### 4.13. Enzyme-Linked Immunosorbent Assay (ELISA) Assay

#### 4.13.1. Indirect ELISA Assay

To determine the soluble tau secreted by different cell cultures, culture supernatants were collected on day 3 after transient transfection. Soluble protein fractions extracted by different cell lysis buffers, RAB, RIAP, or 70% formic acid obtained as described in 4.10, were diluted to a final concentration of 100 μg/mL in PBS. 96 well assay plates were coated with either cell culture supernatants or diluted protein fractions and incubated at 4 °C overnight. Plates were washed 3 times with ELISA wash buffer (Thermo Fisher Scientific) and then blocked with 1% BSA in TBS-T for 1 h. at room temperature (RT). Anti-tau antibody HT7 antibody or anti-tau oligomeric antibody T22 was diluted in blocking buffer (1:2000 in 1% BSA in PBS), added to the plates, and allowed to bind for 2 h. at RT with gentle continual shaking. Plates were then washed three times, followed by the addition of HRP-conjugated goat anti-rabbit IgG (1:2000, Abcam, ab6721) for one hour in the dark at room temperature. Plates were then washed three times with PBS followed by the addition of 1-Step™ Ultra TMB-ELISA Substrate Solution (Thermo Fisher Scientific). Absorbance was detected at 450 nm within 30 min of adding Stop solution on a BioTek Synergy 2 plate reader (Winooski, VT, USA). The total tau concentration in SH-SY5Y cells incubated with different tau-containing exosomes was measured by total human tau ELISA assay (Thermo Fisher Scientific, # KHB0041) following the manufacturer’s instructions.

#### 4.13.2. Sandwich ELISA Assay

To determine oligomeric tau levels, 96 well assay plates were coated with diluted T22 antibody in phosphate buffer pH 7.4 and incubated at 4 °C overnight. ELISA plates were washed once with ELISA wash buffer and blocked with 1% BSA in TBS-T for 1 h at room temperature. RAB or RIPA-extracted soluble protein fractions diluted in PBS were then added and incubated at RT for one hour. Plates were washed three times with ELISA wash buffer followed by the addition of anti-tau antibody HT7 antibody (1:2000) in 1% BSA in PBS for 2 h at RT with gentle continual shaking. Plates were then washed three times followed by the addition of HRP-conjugated goat anti-rabbit IgG (1:2000, Abcam, ab6721) for one hour in the dark at room temperature. Plates were washed three times with PBS followed by the addition of 1-Step™ Ultra TMB-ELISA Substrate Solution (Thermo Fisher Scientific). Absorbance was detected at 450 nm on a BioTek Synergy 2 plate reader within 30 min of adding Stop solution.

### 4.14. Immunocytochemistry

Cell staining was performed as described previously [58]. Briefly, 0.2 × 10^6^ cells were seeded into each well of a 24 well-plate and cultured overnight. The replated cells were then fixed with 4% (*w*/*v*) paraformaldehyde in PBS at room temperature for 15 min and washed twice with a staining buffer (2% (*v*/*v*) FBS in PBS). For staining intracellular markers, the samples were permeabilized with 0.2–0.5% (*v*/*v*) Triton X-100 at room temperature for 15 min. The cells were then blocked with 10% (*v*/*v*) FBS at room temperature for 30 min and then incubated with primary antibodies, including anti-phospho-Tau (Ser199) antibodies, (pS199, Life Technologies, 44734G), anti-phospho-Tau (Ser202, Thr205) antibody (AT8, Life Technologies, MN1020), anti-Tau (phospho T231) antibody (T231, Abcam, ab151559), anti-tau antibody (TAU-5, Life Technologies, MA512808), anti-tau antibody (HT7, Life Technologies, MN1000), anti-tau oligomeric antibody (T22, Millipore Sigma, ABN454), or anti-β-tubulin III antibody (Abcam, ab18207) at room temperature for 4 h. After washing, the cells were incubated with the corresponding secondary antibody: Alexa Fluor^®^ 594 goat anti-Mouse IgG1 (Life Technologies, A21125) or Alexa Fluor^®^ 488 goat anti-Rabbit IgG H&L (Abcam, ab150077), at room temperature for 1 h. The samples were counterstained with Hoechst 33342 (Abcam, ab228551) and visualized using fluorescence microscopy (Keyence BZ-X810).

### 4.15. Flow Cytometry

To quantify the level of microtubule and tau protein expression, the cells were harvested by trypsinization and analyzed by flow cytometry. Briefly, 1 × 10^6^ cells per sample were fixed with 4% paraformaldehyde and washed with staining buffer (2% FBS in PBS). The cells were permeabilized with 100% cold methanol, blocked, and then incubated with primary antibodies against β-tubulin III antibody (Abcam, ab18207) and anti-tau antibody (Tau-5, Life Technologies, MA512808) followed by the corresponding secondary antibodies (Supplementary Table S1). Fluorescence histograms of cells were acquired with a NovoCyte flow cytometer (ACEA Biosciences) and analyzed against isotype controls using FlowJo software.

### 4.16. In-Gel Tryptic Digestion and LC-MS/MS Analysis

For tau phosphorylation determination, approximately 10 µg of *E. coli*-derived purified recombinant tau protein was resolved in multiple lanes by SDS-PAGE. After Coomassie staining, each of the bands was carefully excised with a clean razor blade and the gel slices were shipped to Creative Proteomics (Shirley, NY, USA) for tryptic digestion and LC/MS-MS analysis, performed in triplicate. Greater than 95% coverage of the tau protein sequence was obtained and phosphorylation at each site was determined based on the presence in individual peptides compared with all possible peptides.

### 4.17. Statistical Analysis

Statistical significance was determined by unpaired *t*-tests, one-way analysis of variance (ANOVA), or two-way ANOVA when appropriate using Prism 8 software (GraphPad Software, San Diego, CA, USA). A five percent cutoff was applied to determine statistical significance, and a *p*-value of <0.05 is denoted with one asterisk (*) in relevant figures. In all cases, at least three independent biological replicates from different days were performed and the standard error of the mean is shown.

## 5. Conclusions

**Figure 12:**
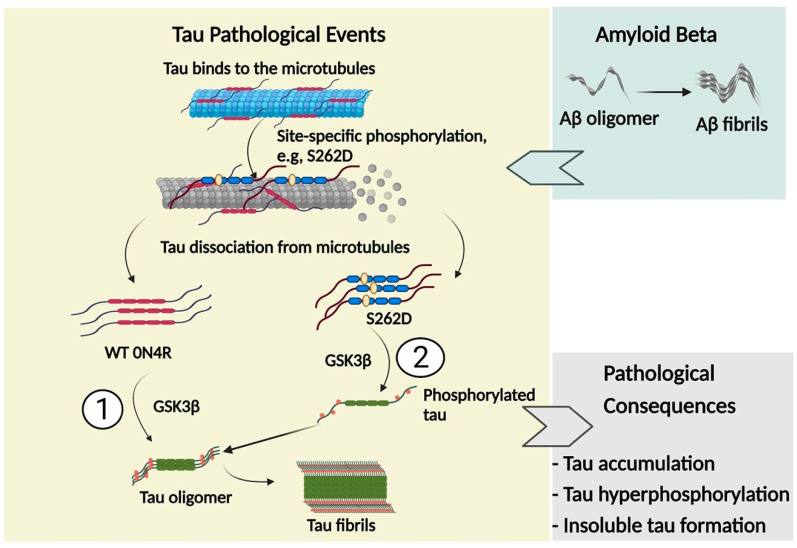
Tau intracellular aggregation and fibril formation appears to result from a series of temporally ordered phosphorylation steps. Site-specific phosphorylation at Ser262 leads to the dissociation of tau from microtubules due to the reduced affinity for microtubules. Addition of Aβ42 oligomers facilitates Ser262 phosphorylation, which may accelerate tau pathology. Cellular tau that is dissociated from microtubules is more accessible to tau kinase GSK3β, which phosphorylates tau further at AD-related sites, such as AT8+ targeted epitopes. Eventually, tau hyper-phosphorylation leads to increased insoluble tau formation. We also observed the activity of GSK3β highly depends on the substrate and can follow two paths to form fibrils (1) GSK3β facilitates tau oligomerization when phosphorylating WT 0N4R. (2) The phosphorylation of tau variant, S262D, can lead to insoluble tau formation Created with BioRender.com; last accessed on 29 August 2022.

In this study, the pathological roles of phosphorylation in tau propagation were investigated. First, we find that GSK3β-induced phosphorylation contributes to every step of tau propagation. GSK3β-induced phosphorylation promoted tau oligomerization and secretion, as well as tau internalization by recipient cells, suggesting a pathological role in tau transmission. Phosphorylated tau showed a much higher propensity to induce intracellular aggregation. Overall, vesicle-free or exosomal tau, which are phosphorylated by GSK3β, showed higher transmissibility than tau with a reduced degree of phosphorylation. Moreover, we found that tau pseudo-phosphorylated at Ser262 showed a weaker affinity to microtubules, disrupted the stability and organization of microtubules, and led to abnormal tau accumulation and further phosphorylation. Most importantly, we found GSK3β acted differently with different tau variants, S262A vs. S262D. Co-transfection of GSK3β further enhanced tau accumulation and led to the insoluble tau formation in S262D tau-overexpressing cells. Finally, we found that pre-treatment of cells with Aβ42 oligomers further exacerbated the pathological consequences caused by overexpressing the S262D tau variant. Taken together, we suggest that a temporally ordered phosphorylation process may be initiated by the pathological hallmark of AD, Aβ42 oligomers, followed by S262 phosphorylation, as illustrated in Figure 12. Following S262 phosphorylation, tau dissociated from microtubules appears to be more accessible to modification by tau kinases including GSK3β for further phosphorylation at multiple sites, leading to insoluble tau accumulation and tau pathology.

## Data Availability

Data is contained within the article or Supplementary Materials.

## Supplementary Materials

The following supporting information can be downloaded at: https://www.mdpi.com/article/10.3390/ijms231911610/s1. Figure S1. The biochemical characterization of HEK293 cells expressing wild type 0N4R and GSK3β constructs. Figure S2. The tau seeding activity of tau-tau sfCherry biosensor CHO K1 cells induced by 1 μM K18 fibrils. Figure S3. Direct mixing tau-expressing HEK293 cells with FRET tau biosensor cells. Figure S4. HEK293 cells with the incubation of exosomes isolated from tau-expressing cells. Figure S5. A higher amount of Tau-5 and β-tubulin III expressions showed in RA-treated SH-SY5Y cells compared to untreated cells, as quantified by flow cytometry. Figure S6. S262 phosphorylation leads to tau cellular redistribution and tau inclusions in SH-SY5Y cells. Table S1. A list of antibodies.
