# Supplementary material for: The Interplay between GSK3β and Tau Ser262 Phosphorylation during the Progression of Tau Pathology
Source: Int J Mol Sci. 2022 Oct 1;23(19):11610. doi: 10.3390/ijms231911610 (PMC9569960; doi:10.3390/ijms231911610)
Supplement: Supplementary file 1 [file ijms-23-11610-s001.zip › ijms-1926579-supplementary.pdf]

Supplementary materials

The interplay between GSK3β and tau Ser262 phosphorylation during the progression of tau pathology

Liqing Song[1], Daniel E Oseid[2], Evan A. Wells[1], Anne Skaja Robinson[1, *]

[1] Department of Chemical Engineering, Carnegie Mellon University, Pittsburgh, PA, 15213, USA
[2] Tulane Brain Institute, Tulane University, New Orleans, LA, 70118, USA
* Correspondence: anne.robinson@cmu.edu; Tel.: (+1) 412-268-7673 (A.S.R.)

Supplementary Table S1. A list of antibodies

Primary Antibody	Origin/ Isotype	Supplier/ Cat#	Secondary antibody
pS199	Rabbit IgG	Life Technologies, 44734G	Alexa Fluor® 488 goat anti-Rabbit IgG H&L (Abcam, ab150077)
pT231	Rabbit IgG	Abcam, ab151559	Alexa Fluor® 488 goat anti-Rabbit IgG H&L (Abcam, ab150077)
HT7	Rabbit IgG	Life Technologies, MN1000	Alexa Fluor® 488 goat anti-Rabbit IgG H&L (Abcam, ab150077)
T22	Rabbit IgG	Millipore Sigma, ABN454	Alexa Fluor® 488 goat anti-Rabbit IgG H&L (Abcam, ab150077)
Tau-5	Mouse IgG1	Life Technologies, MA512808	Alexa Fluor® 594 goat anti-Mouse IgG1(Invitrogen, A-21125)
β-tubulin	Rabbit IgG	Cell Signaling, 3700T	Alexa Fluor® 488 goat anti-Rabbit IgG H&L (Abcam, ab150077)
AT8	Mouse IgG1	Life Technologies, MN1020	Alexa Fluor® 594 goat anti-Mouse IgG1(Invitrogen, A-21125)
77G7	Mouse IgG1	BioLegend, SIG-39405	Alexa Fluor® 594 goat anti-Mouse IgG1(Invitrogen, A-21125)
6HCLC	Rabbit IgG	Life Technologies, 710080	Alexa Fluor® 488 goat anti-Rabbit IgG H&L (Abcam, ab150077)
MAP2	Mouse IgG1	Millipore Sigma, M1406	Alexa Fluor® 594 goat anti-Mouse IgG1(Invitrogen, A-21125)
β-actin	Mouse IgG	Cell Signaling, 8H10D10	Alexa Fluor® 488 goat anti-Mouse (H+L) (Invitrogen, A-11029)
Syntenin-1	Rabbit IgG	Abcam, ab133267	Alexa Fluor® 488 goat anti-Rabbit IgG H&L (Abcam, ab150077)

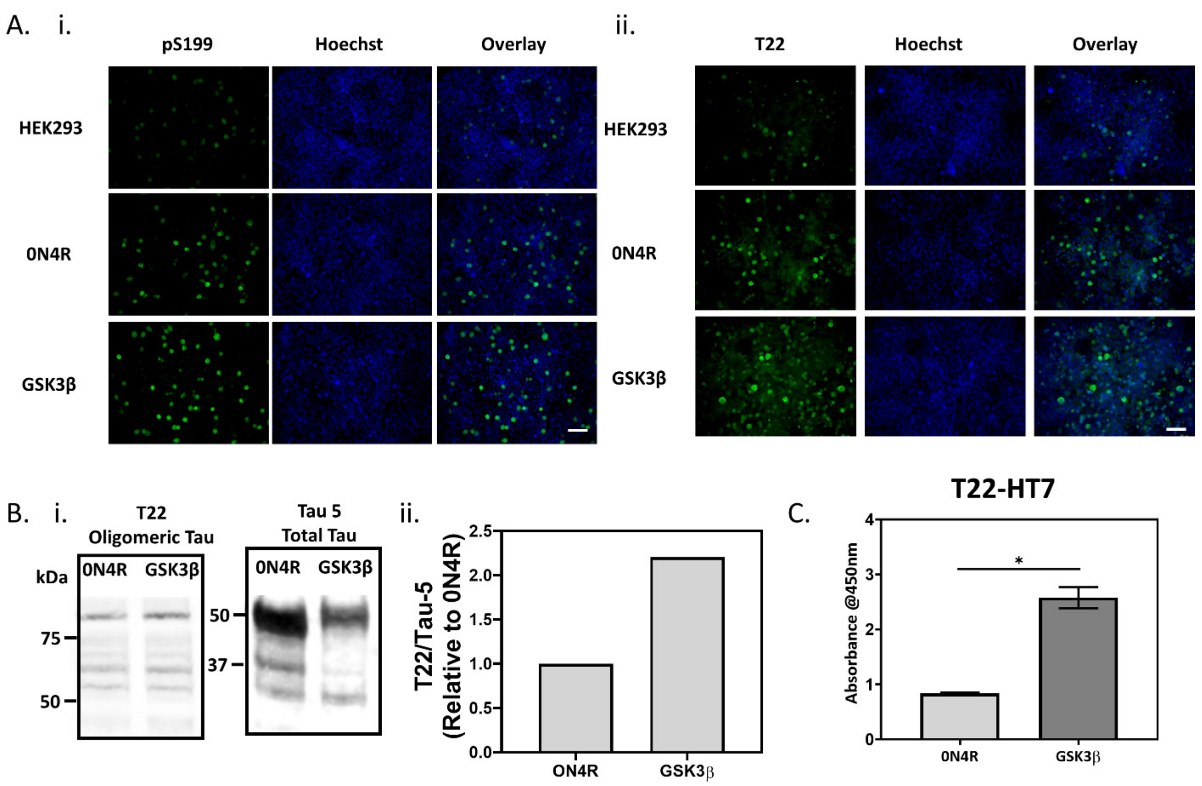

Figure S1. The biochemical characterization of HEK293 cells expressing wild type 0N4R and GSK3β constructs. Representative fluorescent images of tau markers, including pS199 (Green)/Hoechst (Blue) (**A.i.**) and T22(Green)/Hoechst (Blue) **(A.ii.)** for HEK293 cells 3 days post-transfection. Scale bar = 100 µm. Cell lysates from two groups were analyzed by immunoblotting (**B.i.**) against oligomeric tau antibody, T22, and the total tau antibody, Tau-5, followed by quantitation (**B.ii.**) of T22/Tau-5 ratios from a representative western blot experiment. **C.** The oligomeric and total tau were measured by sandwich ELISA using T22 and human tau antibody, HT7, respectively. (* represents $p<0.05$ for 0N4R compared with GSK3β (n=3)).

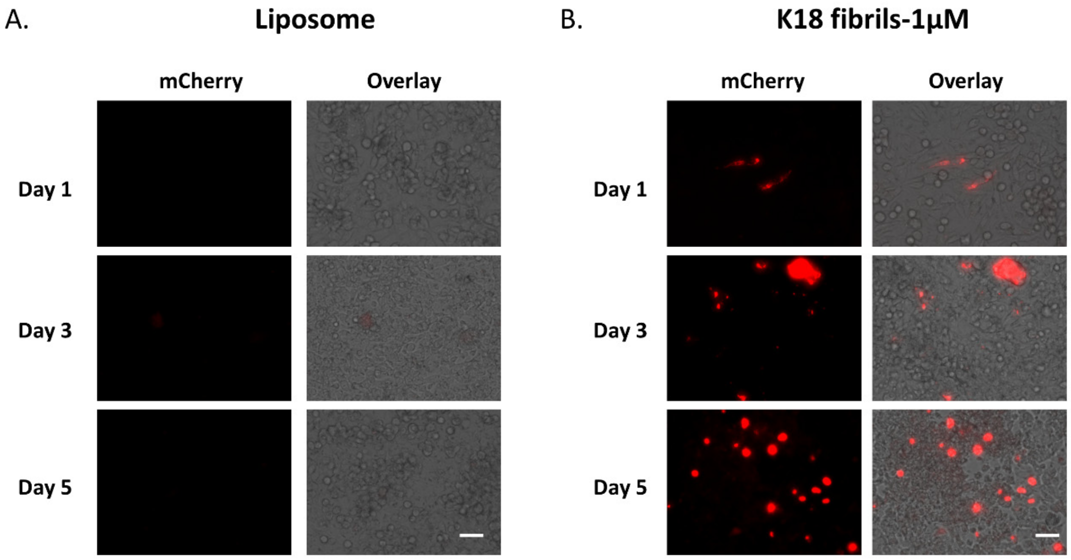

Figure S2. The tau seeding activity of tau-tau sfCherry biosensor CHO K1 cells induced by 1µM K18 fibrils. Fluorescence microscopy images of tau-tau sfCherry biosensor cells treated with 1 µM of K18 tau fibrils for 5 days. Liposome only group showed no red signals. The scale bar denotes 100 µm.

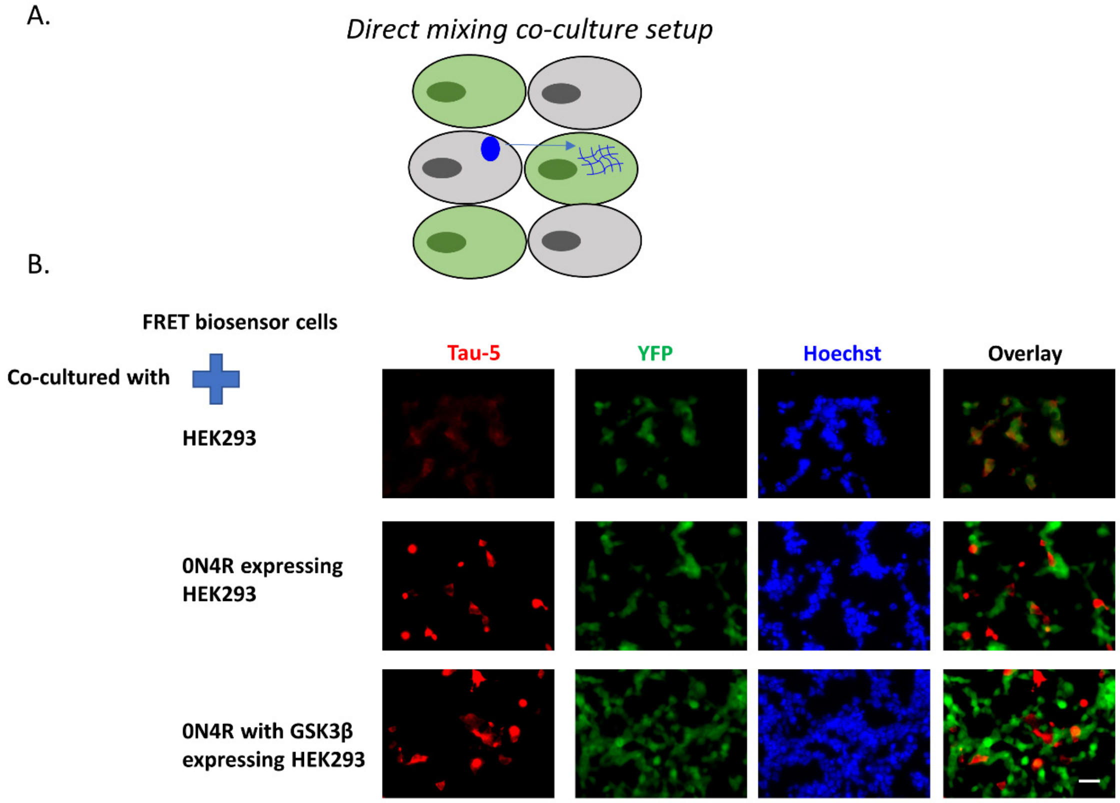

Figure S3 Direct mixing tau-expressing HEK293 cells with FRET tau biosensor cells. A. The schematic illustration of the direct mixing co-culture setup to investigate transcellular tau propagation. **B.** Cells were immunostained against Tau-5 (Red) after three days of co-culturing. The scale bar denotes 100 µm. Transfecting HEK293 cells have indeed increased the amount of Tau-5+ cell population in both 0N4R and 0N4R with GSK3β-expressing HEK293 cells. However, there were no visible tau aggregates formed in tau FRET biosensor cells, indicating the tau species secreted by tau-overexpressing HEK293 cells was incapable of initiating a sequence of events to seed adjacent cells.

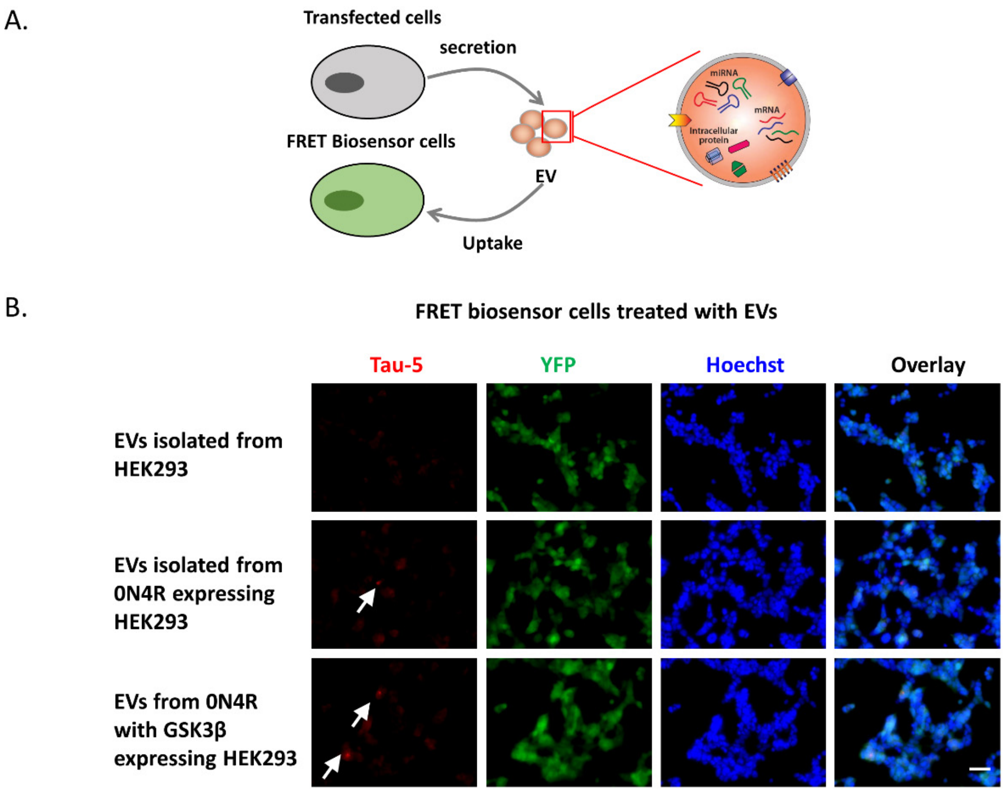

Figure S4. HEK293 cells with the incubation of exosomes isolated from tau-expressing cells. A. The schematic illustration of EV-assisted delivery system to investigate transcellular tau propagation. **B.** HEK293 cells were immunostained against Tau-5 (Red) after three days of EV incubation. The scale bar denotes 100 µm.

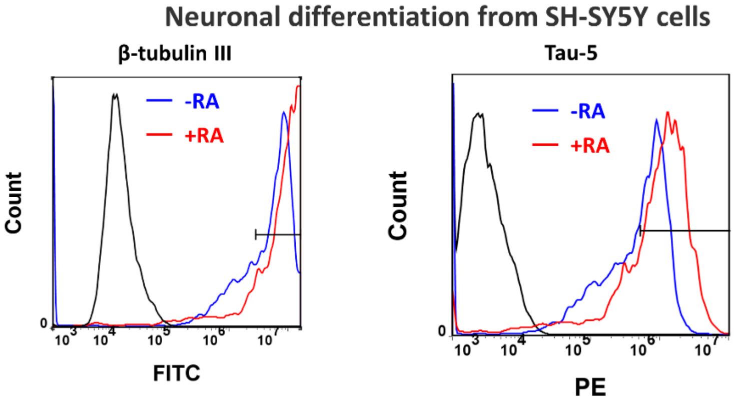

Figure S5. A higher amount of Tau-5 and β-tubulin III expressions showed in RA-treated SH-SY5Y cells compared to untreated cells, as quantified by flow cytometry. Representative flow cytometry histograms are shown. Black line: Negative control; blue line: cells without RA; red line: cells differentiated by RA.

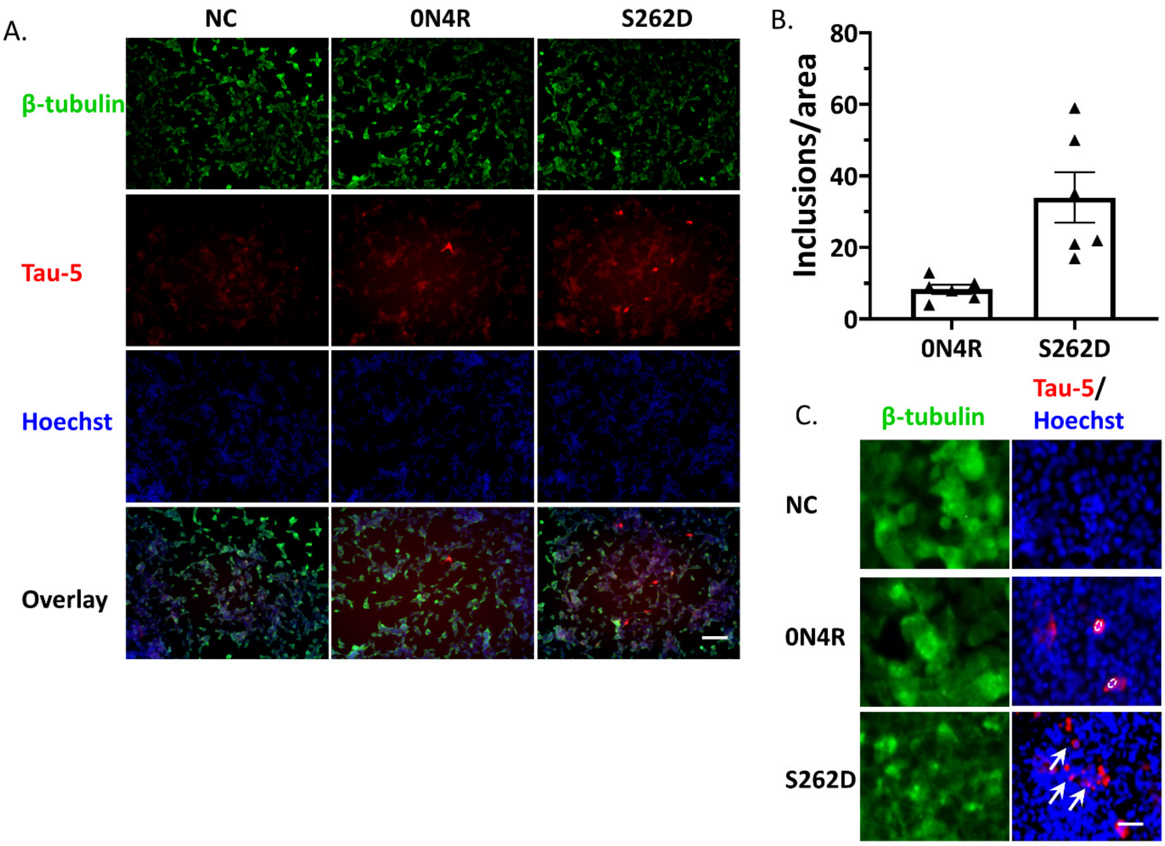

Figure S6. S262 phosphorylation leads to tau cellular redistribution and tau inclusions in SH-SY5Y cells. A. Representative fluorescent images of the neuronal marker, β-tubulin III (Green), and tau marker, Tau-5 (Red), expressed by SH-SY5Y cells transiently transfected with wild-type 0N4R and S262D variant three days following transfection. Negative control (NC): SH-SY5Y cells transfected with pCEP4 plasmid backbone. Scale bar = 100 µm. **B.** Quantifications of the number of Tau-5+ pathological inclusions identified in SH-SY5Y cells three days post-transfection with wild-type 0N4R and S262D variant. **C.** Co-staining of β-tubulin III and Tau-5 showed an overlap in cellular distribution decreased in SH-SY5Y cells transfected with the S262D variant. Scale bar = 25 µm.
